# Prevalence and Intra-Family Phylogenetic Divergence of *Burkholderiaceae*-Related Endobacteria Associated with Species of *Mortierella*

**DOI:** 10.1264/jsme2.ME18081

**Published:** 2018-12-08

**Authors:** Yusuke Takashima, Kensuke Seto, Yousuke Degawa, Yong Guo, Tomoyasu Nishizawa, Hiroyuki Ohta, Kazuhiko Narisawa

**Affiliations:** 1 United Graduate School of Agricultural Science, Tokyo University of Agriculture and Technology 3–5–8 Saiwai-cho, Fuchu, Tokyo 183–8509 Japan; 2 Ibaraki University College of Agriculture 3–21–1 Chuo, Ami, Inashiki, Ibaraki 300–0393 Japan; 3 Mountain Science Center Sugadaira Research Station, University of Tsukuba 1278–294, Sugadaira, Nagano 386–2204 Japan

**Keywords:** *Mortierella*, *Burkholderiaceae*-related endobacteria, *Mycoavidus*, 16S rRNA gene, molecular phylogeny

## Abstract

Endofungal bacteria are widespread within the phylum Mucoromycota, and these include *Burkholderiaceae*-related endobacteria (BRE). However, the prevalence of BRE in Mortierellomycotinan fungi and their phylogenetic divergence remain unclear. Therefore, we examined the prevalence of BRE in diverse species of *Mortierella*. We surveyed 238 isolates of *Mortierella* spp. mainly obtained in Japan that were phylogenetically classified into 59 species. BRE were found in 53 isolates consisting of 22 species of *Mortierella*. Among them, 20 species of *Mortierella* were newly reported as the fungal hosts of BRE. BRE in a *Glomeribacter-Mycoavidus* clade in the family *Burkholderiaceae* were separated phylogenetically into three groups. These groups consisted of a group containing *Mycoavidus cysteinexigens*, which is known to be associated with *M. elongata*, and two other newly distinguishable groups. Our results demonstrated that BRE were harbored by many species of *Mortierella* and those that associated with isolates of *Mortierella* spp. were more phylogenetically divergent than previously reported.

Bacterial endosymbionts are widespread within eukaryotic microorganisms such as algae, protists, and fungi (18, 19, 31, 44). Bacterial endosymbionts in fungi are known as endofungal or endohyphal bacteria, and mostly occur in the phylum Mucoromycota (4, 41). Endofungal bacteria associated with Mucoromycota belonging to the class *Betaproteobacteria* are currently designated as *Burkholderia*-related endobacteria (4) or *Burkholderiaceae*-related endobacteria. For example, *Burkholderia rhizoxinica*, “*Candidatus* Glomeribacter gigasporarum”, and *Mycoavidus cysteinexigens* were found inside the fungal cells of *Rhizopus microsporus* (*Mucorales*, Mucoromycotina), *Gigasporaceae* fungi, such as *Gigaspora margarita* (*Diversisporales*, Glomeromycotina), and *Mortierella elongata* (*Mortierellales*, Mortierellomycotina), respectively (4). These BRE are completely or partly dependent on host nutrients based on a genome analysis (12, 14, 22, 39, 50) and affect gene expression (23, 36, 50), metabolism (23, 26, 35), oxidative stress responses (36, 51), and chemotaxis in their hosts (27).

*Mortierella* spp. inhabit diverse ecological niches such as various soil types (53) and specific substrates, including the bodies of arthropods (8, 9, 54) and animal dung (7, 10, 11, 13, 20). The genus *Mortierella* is one of the largest genera in Mucoromycota and contains nearly 100 described species, which are morphologically classified into nine sections (13) and phylogenetically classified into seven phylogenetic groups (53). Therefore, *Mortierella* spp. have potential as a suitable research material that offers a wider evolutionary perspective on the relationship between BRE and Mucoromycota. Nevertheless, BRE associated with isolates of *Mortierella* spp. were only reported from limited species such as *M. elongata* and *M. minutissima* obtained from soil in Japan and the United States (32, 37, 50).

The objective of the present study was to assess the prevalence of BRE in diverse species of *Mortierella* possessing different properties. We herein examined the presence/absence of BRE within 238 isolates of *Mortierella* spp. mainly obtained in the present study, which were phylogenetically identified as 59 fungal species. We identified many species of *Mortierella* as new fungal hosts of BRE and demonstrated the phylogenetic divergence of BRE associated with isolates of *Mortierella* spp. by a phylogenetic analysis of the 16S rRNA gene. The localization of BRE in fungal host cells was confirmed in twelve isolates consisting of eight species of *Mortierella* by fluorescence and transmission electron microscopic observations.

## Materials and Methods

### Fungal materials

Between 2011 and 2016, we collected five types of substrates, including the dead bodies and dung of animals, fruiting bodies of fungi, fresh and decayed plant materials, and soil from 62 different locations in Japan (Table S1). Estimated temperature data for each geographic coordinate of the collection site were obtained from the WorldClim version 1.4 at 2.5 min of latitude/longitude degree spatial resolution data (15) and putative climates of each collection site were defined by the latitudes and mean temperatures of the warmest and coldest quarters, including 7, 40, and 15 locations in cool, temperate, and subtropical regions, respectively (Table S1). *Mortierella* spp. were isolated using a moist chamber method and inoculating intact or surface-washed substrates onto artificial media. The sporangia of *Mortierella* spp. on substrates or substrate-inoculated media were collected using a frame-sterilized fine needle and inoculated onto fresh _LC_A media (0.2 g yeast extract [Difco, Sparks, MD, USA], 1.0 g glucose [Wako Pure Chemical Industries, Osaka, Japan], 2.0 g NaNO_3_ [Wako], 1.0 g KH_2_PO_4_ [Wako], 0.2 g KCl [Wako], 0.2 g MgSO_4_·7H_2_O [Wako], and 15 g Bacto agar [Difco] in 1.0 L distilled water) (28). In addition, eleven and twelve isolates of *Mortierella* spp. were obtained from the NITE Biological Resource Center (NBRC) and CBS-KNAW culture collection, respectively (Table S1 and S2).

### DNA extraction, PCR, and sequencing of internal transcribed spacer (ITS) regions of *Mortierella* isolates

The nucleotide sequences of the ITS1-5.8S-ITS2 region of each isolate were determined for molecular identification. Template DNA was extracted from mycelia using Prepman^TM^ Ultra sample reagent (Applied Biosystems, Foster City, CA, USA) in accordance with Sato *et al.* (37). Fifty microliters of a PCR mixture containing 1.0 μL of template DNA, 2.5 μL of each forward and reverse fungal universal primer solution (10 pmol μL^−1^ each), 4.0 μL of the dNTP mixture (2.5 mM each), 0.15 μL of 5 U μL^−1^ Ex Taq HS polymerase (Takara Bio, Otsu, Japan), 5.0 μL of 20 mM 10×Taq Buffer, and 34.85 μL of sterilized deionized water was prepared. PCR amplification was performed as follows: initially 5 min for 95°C, followed by 30 cycles of 95°C for 30 s, 54°C for 30 s, and 72°C for 1.5 min, and a final extension step at 72°C for 10 min using a thermal cycler. Forward and reverse primer combinations, such as ITS5-ITS4, ITS1F-ITS4, and ITS5-LR5, were used for PCR (Table S3). PCR products were purified using polyethylene glycol and ethanol precipitation, and a cycle sequence reaction was then performed with a BigDye Terminator Cycle Sequencing Ready Reaction Kit (Applied Biosystems) in accordance with the manufacturers’ instructions with the forward primer used for PCR. Additionally, cycle sequence reactions were also conducted using the reverse primer if needed, and ITS3 and LR0R were used as additional sequencing primers for PCR products using ITS5-LR5 primer combinations (Table S3). Cycle sequencing products were purified by ethanol precipitation, and electrophoresis was performed using an Applied Biosystems 3130xl genetic analyzer (Applied Biosystems) to determine nucleotide sequences. The sequences obtained from each primer were assembled to a single sequence using GeneStudio Professional software version 2.2.0.0 (www.genestudio.com).

### Trimming of large and small subunit coding regions

The sequences obtained from each isolate varied in length because of the different primer combinations and included unnecessary regions for the blastn search at both ends, namely, the large subunit (LSU) and small subunit (SSU) regions. In order to trim off these regions, the start and end positions of the ITS1-5.8S-ITS2 region were detected using ITSx version: 1.0.11 (3). The LSU and/or SSU regions were trimmed off manually from each sequence if present.

### Blastn searches using the ITS1-5.8S-ITS2 region

The genus *Mortierella* is known as one of the most frequently recovered fungal genera in environmental sequencing studies of soil, and there are a large number of environmental and unidentified ITS1-5.8S-ITS2 sequences deposited in GenBank (30). These “unidentifiable” ITS1-5.8S-ITS2 sequences of *Mortierella* often complicate the identification of *Mortierella* spp. using web-based blastn searches. In order to facilitate the identification of the closest species by a blastn search, we constructed a nucleotide sequence database (hereinafter referred to as the custom database) by collecting ITS1-5.8S-ITS2 sequences of Mortierellomycotinan fungi determined using living cultures deposited in culture collections and specimens deposited in herbaria. The custom database consisted of 307 sequences from different phylotypes of 90 Mortierellomycotinan species. Most sequences (299 sequences) were used in a comprehensive phylogenetic analysis of the genus *Mortierella* by Wagner *et al.* (53), and the others were one sequence of *M. elasson* that was obtained from the type strain CBS 220.29, six sequences deposited along with new species descriptions, such as *M. calciphila* (25), *M. fluviae* (16), *M. signyensis* (5), and *M. thereuopodae* (11), and two sequences obtained from *Modicella* spp., from a different genus but phylogenetically included within the genus *Mortierella* (40). The blastn searches with our trimmed ITS1-5.8S-ITS2 sequences using the custom database were conducted to identify the closest species using BLAST+ version 2.5.0 (6). In these blastn searches, the ITS1-5.8S-ITS2 sequences obtained from four BRE-detected isolates of *M. elongata* (FMR13-2, FMR23-1, FMR23-6, and FMR23-9) described in the previous study were included (37). On the other hand, twelve isolates (CBS 122.71, CBS 130.66, CBS 131.66, CBS 222.35, CBS 277.71, CBS 315.61, CBS 316.61, CBS 443.68, CBS 857.70, CBS 858.70, NBRC 109920, and NBRC 109921) obtained from culture collections were excluded and regarded as having 100% similarity because the ITS1-5.8S-ITS2 sequences of these isolates were contained in the custom database.

### Phylogenetic analyses of *Mortierella* spp

Based on blastn searches, untrimmed ITS sequences were separately clustered in different alignment blocks for each phylogenetic group of *Mortierella* spp. defined by Wagner *et al.* (53). The ITS sequences of the custom database were subsequently clustered and combined with the alignment blocks separately. We then conducted phylogenetic analyses using the alignment blocks to clarify the species-level phylogenetic relationship of isolates within each phylogenetic group. Each alignment block was separately aligned, poorly aligned positions were removed, and they were then used for a phylogenetic analysis by the maximum likelihood (ML) method with RA×ML version 8.1.5 software (43) as previously described by Takashima *et al.* (45).

### Diagnostic PCR to detect endofungal bacteria

Diagnostic PCR targeting the bacterial 16S rRNA gene was conducted to detect the presence/absence of endofungal bacteria. Template DNA samples extracted previously for the molecular identification of *Mortierella* spp. were used. Regarding the PCR amplification of the 16S rRNA gene, 50 μL of a PCR mixture containing 1.0 μL of template DNA, 1.5 μL of each primer solution of the bacterial universal primers 10F and 926R (10 pmol μL^−1^ each) (Table S3), 10 μL of 2 mM dNTPs, 1.0 μL of 1.0 U μL^−1^ KOD FX Neo DNA polymerase (Toyobo, Osaka, Japan), 25 μL of 2×PCR Buffer for KOD FX Neo DNA polymerase, and 10 μL of sterilized deionized water was prepared. PCR amplification was performed as follows: initially 2 min for 94°C, followed by 30 cycles of 98°C for 10 s, 58°C for 30 s, and 68°C for 1 min using a thermal cycler, and PCR amplification was checked by agarose gel electrophoresis.

### DNA sequencing of the 16S rRNA gene of *Burkholderiaceae*-related endobacteria

PCR products of the 16S rRNA gene obtained using 10F-926R primers were purified and initially sequenced using the 10F forward primer as a sequencing primer. If the partial sequences obtained with the 10F primer were close to the sequences of known BRE in Mucoromycota using blastn searches in NCBI (https://www.ncbi.nlm.nih.gov/), additional PCR to determine longer sequences suited for the phylogenetic analysis was conducted. The 10F-1541R or 27F-1492R primer combinations were used in additional PCR (Table S3), which was performed using the same conditions as that with the 10F-926R primer set described above. The PCR products of the 16S rRNA sequences of BRE obtained were purified and used for cycle sequencing with the forward and reverse primers used in PCR and additional sequencing primers, such as 786R, 800F, and 926R (Table S3). The sequences obtained from each primer were assembled to a single sequence using GeneStudio Professional software version 2.2.0.0 (www.genestudio.com).

### Phylogenetic analysis of *Burkholderiaceae*-related endobacteria

In the phylogenetic analysis of BRE, the 16S rRNA gene sequences obtained were aligned with the retrieved 16S rRNA gene sequences from GenBank using MAFFT v7.212 (17). The alignment block obtained was viewed using MEGA 6.06 software (46), and poorly aligned positions at both ends were removed manually. Model selection for maximum likelihood phylogeny was performed in MEGA 6.06. The alignment block was used for the phylogenetic analysis with the maximum likelihood (ML) method using RAxML version 8.1.5 software (43) under the GTRGAMMAI model selected by MEGA 6.06 and bootstrapping (1,000 replicates) with the rapid bootstrap analysis option. The alignment block was also used to cluster 16S rRNA gene sequences into operational taxonomic units (OTUs) using mothur v.1.36.1 (method=average) (38). The threshold was set as 97%, which is a common threshold for 16S rRNA gene sequence similarity and has been widely used for the delineation of species in bacterial classification (42).

### Fluorescence microscopy

In order to observe endofungal bacteria inside fungal cells, fluorescence microscopic observations by nucleic staining using a LIVE/DEAD^®^ BacLight^TM^ Bacterial Viability Kit (Molecular Probes, Eugene, OR, USA) and fluorescence *in situ* hybridization (FISH) were performed. Regarding LIVE/DEAD staining, six BRE-harbored isolates (*M. basiparvispora* E1425, *M. humilis* YTM36, and *M. verticillata* CBS130.66 harbored MorBRE group A; *M. alpina* YTM25 harbored MorBRE group B; *M. elongata* FMR13-2 and YTM18 harbored MorBRE group C) and a non-BRE-harbored isolate *M. basiparvispora* E1439 were used. A staining solution containing 1.5 μL each of SYTO9 and propidium iodide fluorescent nucleic acid stains in 1 mL distilled water was freshly prepared and used for staining by adding approximately 100–200 μL of the staining solution onto fungal mycelia placed on a slide. After waiting a few min for staining, the staining solution was removed using a pipette and mycelia were rinsed with adequate amounts of distilled water. A cover slip was then mounted on stained fungal mycelia and excess water was removed using filter paper. Five BRE-harboring isolates and the non-BRE-harboring isolate *M. sugadairana* YTM39s9 were used for FISH. The BRE-harboring isolates were as follows: *M. elongata* FMR23-6 with MorBRE group A; *Mortierella* sp. “zonata” YTM23 with MorBRE group B; *M. parvispora* E2010s1, *M. sugadairana* YTM39, and *Mortierella* sp. 5 YTM49 with MorBRE group C. Fungal mycelia or germinated sporangiospores were fixed in 400 μL of a 3:1 (v/v) mixture of 10% formalin and phosphate-buffered saline (PBS) at 4°C for 6 h in a 1.5-mL tube following two washing steps with 500 μL of PBS and used for hybridization steps. In each case, after removing PBS, the fixed samples were treated using the following steps if needed. Dehydration was performed by adding 200 μL of 50, 75, and 100% ethanol in this order. After ethanol had evaporated, a proteinase K treatment step was performed by adding 200 μL of 10 μg mL^−1^ proteinase K solution at room temperature (RT) for 10 min. After discarding the solution, the following washing steps were performed by adding 200 μL of PBS for 5 min, 200 μL of 1% Tween20 in PBS for 5 min, and rinsing twice with 200 μL of PBS for 5 min again. Treated and untreated samples were then subjected to a hybridization step with an oligonucleotide probe solution containing the universal bacterial 16S rRNA gene probe EUB338 (5′-GCTGCCTCCCGTAGGAGT-3′) (2), which was labeled at the 5′-end with Cy3 (red) or fluorescein isothiocyanate (FITC, green). The oligonucleotide probe solution was a 1:49 mixture of 50 ng μL^−1^ of the oligonucleotide probe and hybridization buffer (34) with a stringency of 35% formamide. In the hybridization step, 100 μL of hybridization solution was added to samples and the probe was hybridized at 46°C for 1.5 h. After hybridization, samples were carefully washed twice with washing buffer (34). After the washing step, samples were placed on a slide and mounted in Slow Fade Diamond Antifade Mountant with DAPI (Molecular Probes). All fluorescence images were obtained using a fluorescence microscope (BX51, Olympus Corp., Tokyo, Japan) equipped with a digital camera DP25 (Olympus Corp.) or EOS kiss X7i (Canon, Tokyo, Japan).

### Transmission electron microscopy

Transmission electron microscopy (TEM) was performed to confirm the endohyphal features of newly distinguishable BRE groups, such as MorBRE groups B and C. Four BRE-harboring isolates were used: *M. elongata* YTM18 and YTM19 with MorBRE group B; *Mortierella* sp. “zonata” YTM23 and *M. alpina* YTM25 with MorBRE group C. Seven-day-old mycelia incubated on a sterilized cellophane sheet placed onto 1/2 CMMY media (10 g malt extract [Difco], 1.0 g yeast extract [Difco], 8.5 g corn meal agar [Difco], and 7.5 g Bacto agar [Difco] in 1.0 L distilled water) at RT were obtained and embedded in a drop of water agar. Regarding fixation, the agar piece was placed in a 1.5-mL tube containing 2.5% glutaraldehyde in 0.05 M cacodylate buffer (CB), pH 7.2 at RT for 2 h. The fixed agar piece was washed in *ca.* 1 mL of 0.05 M CB three times. The fixed agar piece was then postfixed by adding 750 μL of 1% osmium tetroxide in 0.05 M CB at RT in the dark for 2 h. After removing the solution, the fixed agar piece was washed with *ca.* 1 mL distilled water twice. Following a dehydration step using *ca.* 1 mL of 30, 50, 70, 90, and 95% ethanol once, and 100% ethanol twice for 15 min per step, the agar piece was immersed twice in *ca.* 1 mL of a mixed solution of 100% ethanol and acetone (1:1, v/v) twice, and 1 mL of 100% acetone twice for 15 min per step before being infiltrated once in *ca.* 1 mL of agar low viscosity resin (Agar Scientific, Stansted, Essex, UK) in 100% acetone (1:1, v/v) for 1 h and then infiltrated with *ca.* 1 mL of pure resin for 4 h (resin was replaced once at 2 h). The agar piece infiltrated in resin was polymerized at 60°C for 18 h. Ultrathin sections were prepared with a Leica EM UC7 Ultramicrotome (Leica Microsystems GmbH, Wetzlar, Germany). Sections were picked up on grids and stained with 10-fold diluted TI-Blue solution (Nisshin EM, Tokyo, Japan) for 20 min and 0.4% lead citrate solution (52) for 7 min following rinsing three times with distilled water for each staining step. Sections were observed with a Hitachi H-7700 transmission electron microscope (Hitachi, Tokyo, Japan) at an acceleration voltage of 80 kV.

### Statistical analysis

In order to statistically evaluate whether the properties of isolates of *Mortierella* spp., such as climatic zones, phylogenetic groups, and substrates, affected the prevalence of BRE, a generalized linear model (GLM) was examined in the presence/absence of BRE as a binary response variable (binomial family, logit link function, *n*=238). The GLM of the prevalence of BRE incorporated the effects of climatic zones (cool, temperate, subtropical, and unknown), phylogenetic groups (seven groups), and substrates (animal, dung, fungi, plant, soil, and unknown) as categorical explanatory variables, which were the properties of the isolates listed in Table S1, S2, and S4. Using the GLM, odds ratios (OR) with a 95% confidential interval (CI) of the prevalence of BRE in each climatic zone, phylogenetic group, and substrate were calculated by the function “logistic.display()” in the R package “epiDisplay” (https://CRAN.R-project.org/package=epiDisplay) with comparisons to that in the temperate zone, phylogenetic group 7, and soil as reference levels of each explanatory variable, respectively. The combination of properties of reference levels was the most typical in the isolates examined (35 out of 238 isolates). All statistical analyses were performed using R version 3.3.1 (https://www.r-project.org/).

### Primer information and sequence data

All the details of primer information used in the present study are listed in Table S3. Sequences containing the ITS1-5.8S-ITS2 region were deposited under accession no. MF403050–MF403052, MF510830, and MH802517–MH802523 for the eleven isolates obtained from culture collections, and MF423485–MF423695 for the 211 isolates newly obtained in this study. 16S rRNA gene sequences of BRE were deposited under accession no. MF383419–MF383462 and MH760809–MH760813 for the 49 sequences used in the phylogenetic analysis.

## Results and Discussion

### Relaxed intraspecific host range by *Burkholderiaceae-*related endobacteria associated with *Mortierella* spp

**Fig. 1:**
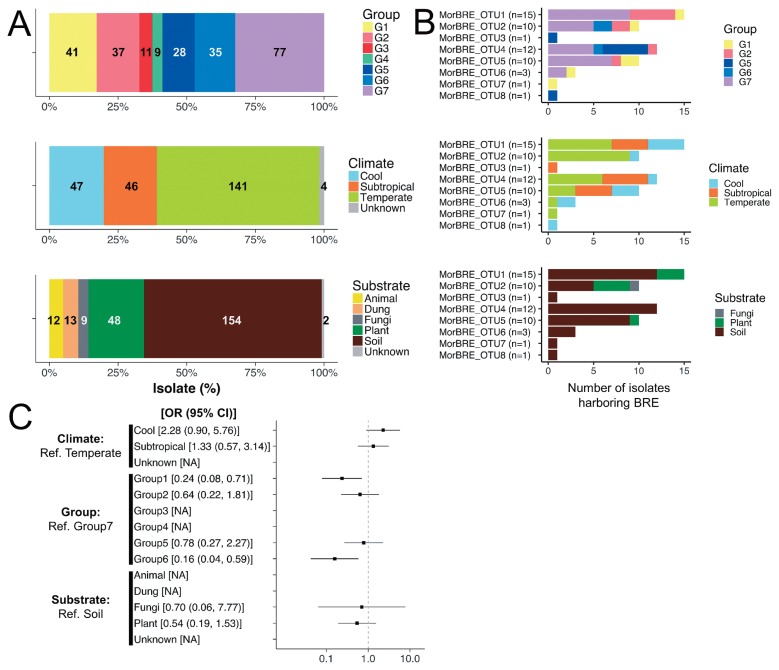
The number of isolates of *Mortierella* examined in the present study (A) and harboring different OTUs of *Burkholderiaceae*-related endobacteria with different properties, such as phylogenetic groups of the fungal host, climatic zones, and substrates (B), and odds ratios showing the effects of the different properties of isolates of *Mortierella* on the prevalence of BRE (C). In graph B, Each OTU was defined by the threshold with 97% similarity to the 16S rRNA gene using mothur v.1.36.1. “MorBRE_OTU1”, “MorBRE_OTU2”, and “MorBRE_OTU3”, and other OTUs (MorBRE_OTU4, 5, 6, 7 and 8) were located in the clades MorBRE groups A, B, and C, respectively, shown in the ML phylogenetic tree. In graph C, the odds ratio (OR) with a 95% confidential interval (CI) was estimated using a generalized linear model (GLM) on the presence/absence of BRE (a binary response variable) incorporating the effects of climatic zones, phylogenetic groups, and substrates as categorical explanatory variables, which were the properties of *Mortierella* isolates (binomial family, logit link function, *n*=238). The prevalence of BRE in each climatic zone, phylogenetic group, and substrate of *Mortierella* isolates was compared to that in the temperate zone, phylogenetic group 7, and soil as a reference level, respectively. “NA” indicates that the odds ratio is not available because the prevalence of BRE was not identified in this study.

Approximately 89% (211 out of 238 isolates) of fungal isolates were newly obtained in the present study. Most of the ITS sequences associated with these isolates (189 out of 226) showed high similarities of more than 97%, which was the threshold in *Mortierella* spp. representing an almost linear relationship between the number of type materials currently sequenced and the number of identifiable molecular OTUs (30). The other ITS sequences showed similarities of less than 97% (Table S4). These 238 isolates were phylogenetically clustered in 59 species, including the 38 described species (Fig. S1 and Table S4), which represent all seven phylogenetic groups of *Mortierella* spp. (Fig. 1A) defined by Wagner *et al.* (53). The number of identified fungal species corresponded to approximately half of the fungal species contained in the custom database.

In the present study, 234 isolates of *Mortierella* spp. were examined for the presence/absence of BRE. Diagnostic PCR targeted to the bacterial 16S rRNA gene revealed that BRE were detected in approximately 22% (53 out of 238) isolates and 37% (22 out of 59) species of *Mortierella* (Table 1). The 22 species of *Mortierella* harboring BRE were classified into five phylogenetic groups defined by Wagner *et al.* (53): Group 1 (six species), Group 2 (four species), Group 5 (five species), Group 6 (one species), and Group 7 (six species) (Table 1). *Mortierella* spp. are traditionally classified into nine sections based on their asexual morphologies, *i.e.*, *Actinomortierella*, *Alpina*, *Haplosporangium*, *Hygrophila*, *Mortierella*, *Schmuckeri*, *Simplex*, *Spinosa*, and *Stylospora* (13). Even though this classification was not supported by the phylogenetic classification (53), similarities in asexual morphologies may represent its reproductive strategies, such as dispersion. Among these nine morphology-based sections, 13 species included in the 22 BRE-harboring species were classified into six sections (*Actinomortierella* [one species], *Alpina* [one species], *Hygrophila* [two species], *Simplex* [one species], *Spinosa* [five species], and *Stylospora* [three species]) (Table 1). Previous studies showed that BRE were associated with only two species, *M. elongata* (phylogenetic group 7, section *Hygrophila*) and *M. minutissima* (phylogenetic group 2, section *Hygrophila*), in *Mortierella* spp. (32, 37, 50). Therefore, these results suggested that BRE associated with phylogenetically and morphologically broad host species in *Mortierella* spp.

### Polyphyly of *Burkholderiaceae*-related endobacteria associated with *Mortierella* spp

**Fig. 2:**
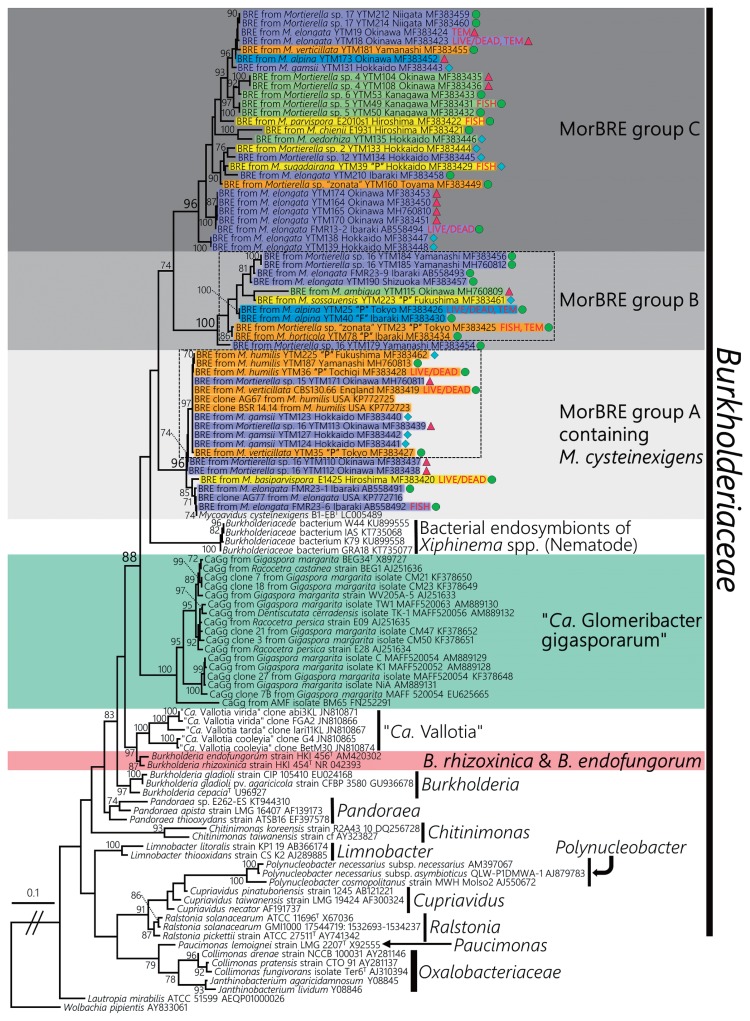
Maximum likelihood (ML) phylogenetic tree of BRE in the family *Burkholderiaceae* based on partial 16S rRNA gene sequences (1334 positions) using RAxML version 8.1.5 software with the GTRGAMMAI model and bootstrapping (1,000 replicates) with the rapid bootstrap analysis option. Bootstrap values >70% are shown at nodes. The value of the log likelihood was −12105.124158. *Wolbachia pipientis* was used as an outgroup. Backgrounds show bacterial clades consisting of BRE. BRE clades associated with *Mortierella* spp. were classified into MorBRE groups A, B, and C. Some subclades including BRE associated with *Mortierella* spp. obtained from plant materials are indicated by boxes with dashed lines. Taxa names of BRE-detected isolates of *Mortierella* are shown with different colored backgrounds corresponding to the phylogenetic groups of *Mortierella*: groups 1 (yellow), 2 (orange), 5 (green), 6 (blue), and 7 (purple) as defined by Wagner *et al.* (53). Isolates of *Mortierella* spp. obtained from plant or fungal materials are indicated with “P” and “F”, respectively, according to the name of the isolate; the absence of these marks indicates that the isolates were obtained from soil. BRE-detected isolates of *Mortierella* spp. used for microscopic observations (LIVE/DEAD staining, FISH, or TEM) are shown in red letters. Diamonds, circles, and triangles at the end of the taxa name indicate the climate at which the hosts were obtained as cool, temperate, and subtropical regions, respectively.

**Fig. 3:**
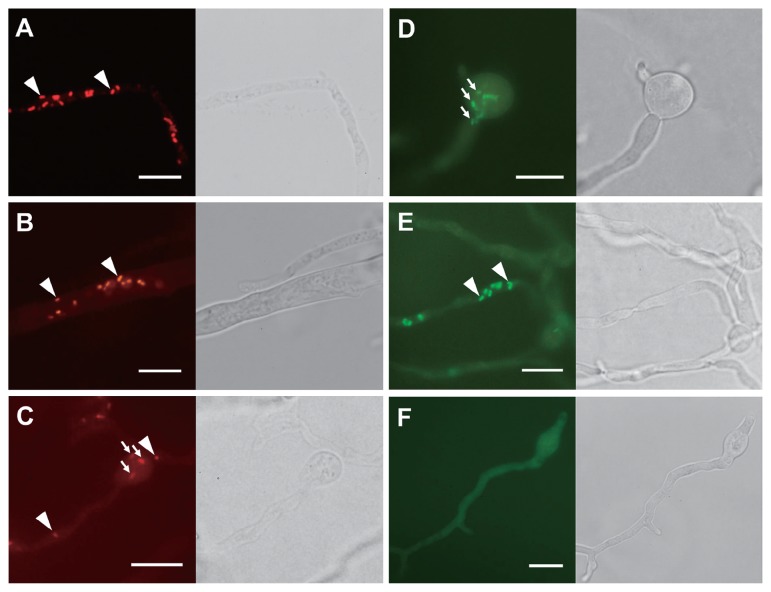
FISH images of BRE-harbored isolates (A–E) and BRE-cured isolate of *Mortierella* (F). Bright field images are shown beside each FISH image. FISH was performed using Cy3-labeled (Red, A–C) or FITC-labeled (Green, D–F) EUB338 probes. Bacterial cells within hyphae and sporangiospores are indicated by arrowheads and arrows, respectively. Rod-shaped endofungal bacterial cells were detected within the hypha of *M. elongata* FMR23-6 (A), *Mortierella* sp. “zonata” YTM23 (B), and *M. sugadairana* YTM39 (E). Rod-shaped endofungal bacterial cells were detected within sporangiospores of *M. parvispora* E2010s1 (C) and *Mortierella* sp. 5 YTM49 (D). No bacterial cells were detected within *M. sugadairana* YTM39s9 (F), which is a BRE-cured isolate of *M. sugadairana* YTM39 (E). Scale bars: 10 μm.

**Fig. 4:**
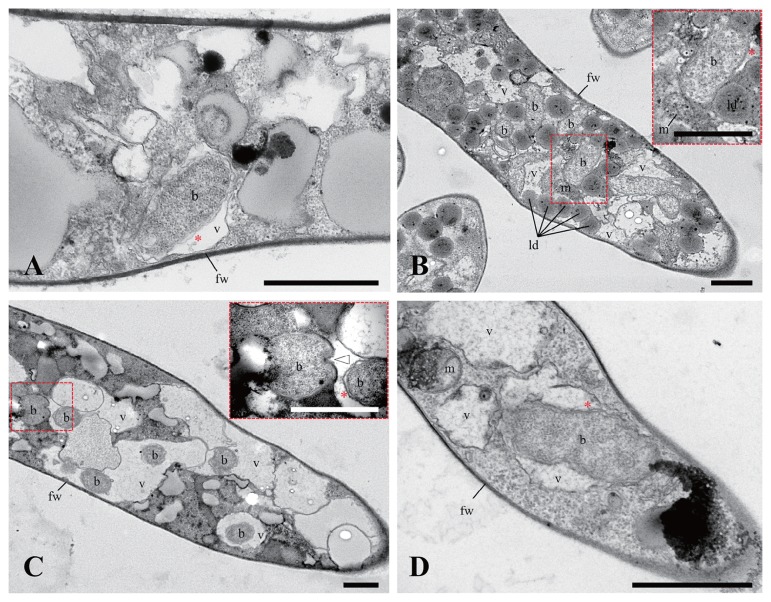
TEM images of BRE-harbored isolates of *Mortierella* (A–D). Bacterial cells were detected within hypha of *Mortierella* sp. “zonata” YTM23 (A), *M. alpina* YTM25 (B), *M. elongata* YTM18 (C), and *M. elongata* YTM19 (D). b, bacterium; fw, fungal cell wall; ld, lipid droplet; v, vacuole; m, mitochondrion; arrowhead, endofungal bacterium showing binary fission; *, bacterial double-layered cell envelope. Scale bars: 1 μm.

A ML phylogenetic tree with 1334 positions showed that all BRE were located in the family *Burkholderiaceae* (Fig. 2). Furthermore, BRE detected from the *Mortierella* spp., “*Ca.* Glomeribacter gigasporarum”, and bacterial endosymbionts associated with the root parasitic nematodes, *Xiphinema* spp. (33), were clustered together in a single clade corresponding to a *Glomeribacter-Mycoavidus* clade (50). This clade was sister to another clade containing BRE in *Rhizopus* spp., such as *B. rhizoxinica* and *B. endofungorum*, and “*Ca.* Vallotia” spp. (47) (Fig. 2). Within the former clade, we found that BRE detected from *Mortierella* spp. were divided into three well-supported clades, identified as MorBRE groups A, B, and C (Fig. 2). By clustering OTUs at a 97% similarity using mothur, 16S rRNA gene sequences located within the MorBRE groups A, B and C were divided into one, two, and five OTUs, respectively (Table 2). A subclade consisting only of “*Ca.* Glomeribacter gigasporarum” and a subclade containing *M. cysteinexigens* (named MorBRE group A in the present study) were previously reported within the *Glomeribacter- Mycoavidus* clade (32, 37, 50); however, the present study found two additional groups within the clade that only consisted of BRE associated with *Mortierella* spp., named MorBRE groups B and C, respectively (Fig. 2). Our microscopic observations showed that these additional BRE groups were harbored in the host cells (MorBRE group B: Fig. 3B, 4A, 4B, and S2C; MorBRE group C: Fig. 3C, 3D, 3E, 4C, 4D, S2A, and S2B) as well as MorBRE group A (Fig. 3A, S2D, S2E, and S2F). In contrast, bacterial cells were not observed in two non-BRE harbored isolates in which endofungal bacteria were not detected by diagnostic PCR (Fig. 3F and S2G). TEM observation clarified that these phylogenetically new BRE were observed as rod-shape cells in a longitudinal section possessing a double-layered cell envelope and cytoplasm rich in ribosomes within the host hypha (MorBRE group B: Fig. 4A and B; MorBRE group C: Fig. 4C and D) similar to those of *M. cysteinexigens* (37, 50) and were frequently observed in vacuoles in the host hypha (Fig. 4). Therefore, these results indicated that phylogenetically different BRE were harbored within the hyphae of *Mortierella* spp. In addition, according to the phylogenetic analysis, the phylogenetic positions of BRE associated with *Mortierella* spp. were not related to the phylogenetic groups of the fungal hosts (Fig. 2). Evidence for phylogenetic co-divergence between BRE and fungal hosts was previously obtained for both associations, *Rhizopus*-*Burkholderia* (21) and *Gigasporaceae*-“*Ca.* Glomeribacter” (29). On the other hand, it currently remains unclear whether BRE associated with isolates of *Mortierella* spp. and its fungal hosts were co-divergent or not. However, according to the ML phylogenetic tree, the transmission of these BRE was not circumscribed only by vertical or horizontal transmission because BRE classified in the same OTUs were sometimes found in several different species of *Mortierella* (Fig. 2 and Table 2). Furthermore, Lastovetsky *et al.* (24) newly detected BRE in *Acaulospora* sp. (*Acaulosporaceae*, *Diversisporales*, and Glomeromycotina) as a new fungal host group of BRE. It was phylogenetically close to *M. cysteinexigens* detected in *M. elongata* FMR23-6. Therefore, the potential for the horizontal transmission of BRE among *Mortierella* spp. and other fungal hosts within subphyla Glomeromycotina exists. In the future, the fungal resources obtained in the present study will contribute to the construction of more robust phylogenies of BRE and its hosts using multi-locus genes and a co-phylogenetic analysis of BRE and its hosts in order to estimate whether BRE is transmitted horizontally among different *Mortierella* spp. and/or among fungal hosts within different subphyla.

### Relationship between abiotic factors and the prevalence of *Burkholderiaceae*-related endobacteria associated with *Mortierella* spp

In the present study, BRE were detected in fungal isolates obtained from soil at 29% (44 out of 154 isolates), plant materials at 17% (8 out of 48 isolates), and fungal materials at 11% (1 out of 9 isolates), but was not detected in fungal isolates originating from animal materials, such as bodies and dung (Fig. 1B). BRE were detected in fungal isolates obtained from various locations in different climates in Japan (Table S1 and S2). The prevalence rates of BRE were 30% (14 out of 46 isolates), 26% (12 out of 47 isolates), and 19% (27 out of 141 isolates) in subtropical, cool, and temperate climatic zones, respectively (Fig. 1B and Table 1). Differences in the prevalence of bacterial endosymbionts with a focus on geographical, climatic, and ecological factors are often suggested in arthropod hosts (*e.g.* 1, 48, 49); however, this has not been attempted for the bacterial endosymbionts of fungi. Based on the results of our examination of BRE, we initially attempted to evaluate whether abiotic factors (the properties of isolates of *Mortierella* spp.) affected the prevalence of BRE in hosts by calculating OR with a 95% CI using a GLM incorporating the effects of abiotic factors, such as climatic zones, phylogenetic groups, and substrates for categorical explanatory variables. The results obtained showed that most of the differences in the properties of isolates of *Mortierella* spp. did not appear to affect the prevalence of BRE because most of the ORs of each variable included OR=1 (Fig. 1C). However, CI values were wide because the number of isolates harboring BRE was small (Fig. 1C). Therefore, extensive examinations on the selected *Mortierella* spp., such as *M. elongata*, obtained from BRE-harbored isolates from different climatic zones will contribute to more accurate investigations on the effects of environmental factors on the presence/absence of BRE.

### An evolutionary perspective on *Burkholderiaceae*-related endobacteria associated with *Mortierella* spp

The phylogenetic positions of BRE associated with *Mortierella* spp. did not appear to be circumscribed by the species of fungal hosts or climatic zones of the fungal hosts isolated (Fig. 2). On the other hand, we found that the phylogenetic positions of some BRE taxa detected in isolates obtained from plant materials mainly clustered into two different clades located in MorBRE groups A and B, respectively (boxes with a dashed line in Fig. 2). Since the number of BRE detected in isolates obtained from plant materials was limited, this result implies that the phylogenetic positions of BRE associated with isolates of *Mortierella* spp. are circumscribed by the isolation substrates of the hosts. Palomares-Rius *et al.* (33) previously reported a phylogenetic relationship between BRE and bacterial endosymbionts associated with the plant root parasitic nematodes, *Xiphinema* spp. Our phylogenetic tree emphasized that the clade containing the bacterial endosymbionts of nematodes was related to the *Glomeribacter-Mycoavidus* clade and showed that the clade was a sister to clades of BRE associated with *Mortierella* spp.

## Supplemental Material



## Figures and Tables

**Table 1:** Presence/absence of *Burkholderiaceae*-related endobacteria (BRE) in isolates of *Mortierella* obtained from different climatic zones.

Species identified by integrating blastn searches and phylogenetic analyses*	No. of isolates harbored BRE/total no. of isolates	Presence of BRE	Prevalence of BRE in each phylogenetic group of hosts	No. of isolates harboring BRE/total no. of isolates obtained from different climates			

				Cool	Temperate	Subtropical	Unknown
*M. alliacea*^b^	0/1	−	Group 1, 15% (6/41 isolates)	0/1	—	—	—
*M. chienii*^h^	1/12	+		—	1/11	0/1	—
*M. cystojenkinii*^h^	0/1	−		—	0/1	—	—
*M. pulchella*^h^	0/2	−		0/2	—	—	—
*M. selenospora*^d^	0/2	−		—	—	0/2	—
*M. sossauensis*^h^	1/2	+		1/2	—	—	—
*M. basiparvispora*^d^	1/3	+		0/1	1/2	—	—
*M. jenkinii*^h^	0/1	−		—	0/1	—	—
*M. parvispora*^h^	1/10	+		0/5	1/5	—	—
*M. sugadairana*^h^	1/3	+		1/3	—	—	—
*Mortierella* sp. 1†	0/2	−		0/2	—	—	—
*Mortierella* sp. 2†	1/1	+		1/1	—	—	—
*Mortierella* sp. 3†	0/1	−		—	—	0/1	—

*M. clonocystis*^d^	0/3	−	Group 2, 24% (9/37 isolates)	0/3	—	—	—
*M. epicladia*^h^	0/1	−		—	—	0/1	—
*M. humilis*^i^	3/10	+		1/3	2/6	—	0/1
*M. verticillata*^i^	3/7	+		0/2	3/5	—	—
*M. horticola*^i^	1/10	+		—	1/10	—	—
*M. minutissima*^d^	0/4	−		—	0/4	—	—
*Mortierella* sp. “zonata”†	2/2	+		—	2/2	—	—

*M. calciphila*^d^	0/3	−	Group 3, 0% (0/11 isolates)	—	0/3	—	—
*M. gemmifera*^d^	0/5	−		0/5	—	—	—
*M. kuhlmanii*^d^	0/3	−		—	0/3	—	—

*Dissophora decumbens*†	0/1	−	Group 4, 0% (0/9 isolates)	0/1	—	—	—
*Gamsiella multidivaricata*†	0/1	−		—	0/1	—	—
*M. globulifera*^g^	0/7	−		0/1	0/4	—	0/2

*M. ambigua*^a^	1/1	+	Group 5, 25% (7/28 isolates)	—	—	1/1	—
*M. capitata*^a^	0/10	−		—	0/10	—	—
*M. oedorhiza*^g^	1/1	+		1/1	—	—	—
*M. wolfii*^h^	0/2	−		—	0/2	—	—
*Mortierella* sp. 4†	2/4	+		—	—	2/4	—
*Mortierella* sp. 5†	2/3	+		—	2/3	—	—
*Mortierella* sp. 6†	1/1	+		—	1/1	—	—
*Mortierella* sp. 7†	0/1	−		—	0/1	—	—
*Mortierella* sp. 8†	0/4	−		—	0/1	0/3	—
*Mortierella* sp. 9†	0/1	−		—	—	0/1	—

*M. alpina*^b^	3/21	+	Group 6, 9% (3/35 isolates)	0/1	2/14	1/6	—
*M. hypsicladia*^h^	0/1	−		—	0/1	—	—
*M. oligospora*^e^	0/1	−		—	0/1	—	—
*M. polycephala*^e^	0/2	−		0/1	0/1	—	—
*Mortierella* sp. 10†	0/7	−		—	—	0/7	—
*Mortierella* sp. 11†	0/3	−		0/3	—	—	—

*M. biramosa*^e^	0/2	−	Group 7, 36% (28/77 isolates)	—	—	0/2	—
*M. elongata*^d^	14/34	+		2/3	6/25	6/6	—
*M. exigua*^h^	0/1	−		—	—	0/1	—
*M. fatshederae*^d^	0/1	−		—	—	0/1	—
*M. gamsii*^h^	4/4	+		4/4	—	—	—
*M. thereuopodae*^h^	0/2	−		—	0/2	—	—
*M. zonata*^i^	0/1	−		—	0/1	—	—
*M. zychae*^d^	0/4	−		—	0/4	—	—
*Mortierella* sp. CBS 118520†	0/1	−		—	0/1	—	—
*Mortierella* sp. 12†	1/3	+		1/2	—	—	0/1
*Mortierella* sp. 13†	0/4	−		—	0/4	—	—
*Mortierella* sp. 14†	0/1	−		—	0/1	—	—
*Mortierella* sp. 15†	1/6	+		—	0/1	1/5	—
*Mortierella* sp. 16†	6/7	+		—	3/4	3/3	—
*Mortierella* sp. 17†	2/3	+		—	2/3	—	—
*Mortierella* sp. 18†	0/1	−		—	—	0/1	—
*Mortierella* sp. 19†	0/2	−		—	0/2	—	—

Prevalence of BRE	22% (53/238 isolates)	37% (22/59 species)		26% (12/47 isolates)	19% (27/141 isolates)	30% (14/46 isolates)	0% (0/4 isolates)

* Sections based on asexual morphologies were indicated for each name as follows:

a *Actinomortierella*,

b *Alpina*,

c *Haplosporangium*,

d *Hygrophila*,

e *Mortierella*,

f *Schmuckeri*,

g *Simplex*,

h *Spinosa*,

i *Stylospora*,

† Not designated.

**Table 2:** Phylogenetic positions of *Burkholderiaceae*-related endobacteria (BRE) associated with isolates of *Mortierella*.

Fungal host		*Burkholderiaceae*-related endobacteria		
	
Host species identified by integrating blastn searches and phylogenetic analyses	Isolate no. of fungal hosts	Placement of phylogenetic clades of detected BRE	OTUs of BRE classified by mothur	Accession no. of 16S rRNA genes of detected BRE
*M. basiparvispora*	E1425	A	OTU1	MF383420
*M. elongata*	FMR23-1	A	OTU1	AB558491
*M. elongata*	FMR23-6	A	OTU1	AB558492
*M. gamsii*	YTM123	A	OTU1	MF383440
*M. gamsii*	YTM124	A	OTU1	MF383441
*M. gamsii*	YTM127	A	OTU1	MF383442
*M. humilis*	YTM36	A	OTU1	MF383428
*M. humilis*	YTM187	A	OTU1	MH760813
*M. humilis*	YTM225	A	OTU1	MF383462
*M. verticillata*	YTM35	A	OTU1	MF383427
*M. verticillata*	CBS 130.66	A	OTU1	MF383419
*Mortierella* sp. 15	YTM171	A	OTU1	MH760811
*Mortierella* sp. 16	YTM110	A	OTU1	MF383437
*Mortierella* sp. 16	YTM112	A	OTU1	MF383438
*Mortierella* sp. 16	YTM113	A	OTU1	MF383439
*M. alpina*	YTM25	B	OTU2	MF383426
*M. alpina*	YTM40	B	OTU2	MF383430
*M. elongata*	FMR23-9	B	OTU2	AB558493
*M. elongata*	YTM190	B	OTU2	MF383457
*M. horticola*	YTM78	B	OTU2	MF383434
*M. sossauensis*	YTM223	B	OTU2	MF383461
*Mortierella* sp. “zonata”	YTM23	B	OTU2	MF383425
*Mortierella* sp. 16	YTM179	B	OTU2	MF383454
*Mortierella* sp. 16	YTM184	B	OTU2	MF383456
*Mortierella* sp. 16	YTM185	B	OTU2	MH760812
*M. ambigua*	YTM115	B	OTU3	MH760809
*M. alpina*	YTM173	C	OTU4	MF383452
*M. elongata*	YTM18	C	OTU4	MF383423
*M. elongata*	YTM19	C	OTU4	MF383424
*M. gamsii*	YTM131	C	OTU4	MF383443
*M. verticillata*	YTM181	C	OTU4	MF383455
*Mortierella* sp. 4	YTM104	C	OTU4	MF383435
*Mortierella* sp. 4	YTM108	C	OTU4	MF383436
*Mortierella* sp. 5	YTM49	C	OTU4	MF383431
*Mortierella* sp. 5	YTM50	C	OTU4	MF383432
*Mortierella* sp. 6	YTM53	C	OTU4	MF383433
*Mortierella* sp. 17	YTM212	C	OTU4	MF383459
*Mortierella* sp. 17	YTM214	C	OTU4	MF383460
*M. elongata*	FMR13-2	C	OTU5	AB558494
*M. elongata*	YTM164	C	OTU5	MF383450
*M. elongata*	YTM165	C	OTU5	MH760810
*M. elongata*	YTM170	C	OTU5	MF383451
*M. elongata*	YTM174	C	OTU5	MF383453
*M. elongata*	YTM210	C	OTU5	MF383458
*M. sugadairana*	YTM39	C	OTU5	MF383429
*Mortierella* sp. 2	YTM133	C	OTU5	MF383444
*Mortierella* sp. 12	YTM134	C	OTU5	MF383445
*Mortierella* sp. “zonata”	YTM160	C	OTU5	MF383449
*M. elongata*	YTM138	C	OTU6	MF383447
*M. elongata*	YTM139	C	OTU6	MF383448
*M. parvispora*	E2010s1	C	OTU6	MF383422
*M. chienii*	E1931	C	OTU7	MF383421
*M. oedorhiza*	YTM135	C	OTU8	MF383446
