# Supplementary material for: Prevalence and Intra-Family Phylogenetic Divergence of Burkholderiaceae-Related Endobacteria Associated with Species of Mortierella
Source: Microbes Environ. 2018 Dec 8;33(4):417–27. doi: 10.1264/jsme2.ME18081 (PMC6307997; doi:10.1264/jsme2.ME18081)
Supplement: Supplementary file 1 [file 33_417_s1.pdf]

Supplemental Material

Title: Prevalence and Intra-Family Phylogenetic Divergence of *Burkholderiaceae*-Related Endobacteria Associated with Species of *Mortierella*

Authors and affiliations:

Yusuke Takashima[1, 2], Kensuke Seto[3], Yousuke Degawa[3], Yong Guo[2], Tomoyasu Nishizawa[1, 2], Hiroyuki Ohta[1, 2], Kazuhiko Narisawa[1, 2, *]

1 United Graduate School of Agricultural Science, Tokyo University of Agriculture and Technology, 3-5-8 Saiwai-cho, Fuchu, Tokyo 183-8509, Japan

2 Ibaraki University College of Agriculture, 3-21-1 Chuo, Ami, Inashiki, Ibaraki, 300-0393, Japan

3 Mountain Science Center Sugadaira Research Station, University of Tsukuba, 1278-294, Sugadaira, Nagano 386-2204, Japan

*Corresponding author: Kazuhiko Narisawa

Tel: +81 29-888-8667

Fax: +81 29-888-8667

Email: kazuhiko.narisawa.kkm@vc.ibaraki.ac.jp

Topic: Microbial interactions and interrelations with other organisms (symbiosis and pathogenesis for plants, animals, and human)

Running headline: Endofungal bacteria in *Mortierella*

Table S1

List of collection sites in this study and their estimated temperature data obtained from the WorldClim version 1.4.

Table S2

List of isolates of *Mortierella* spp. used in this study.

Table S3

List of primers used in this study.

Table S4

Phylogenetically identified species of isolates used in this study based on blastn searches and phylogenetic analyses using ITS sequences.

Figure S1

Unrooted maximum likelihood (ML) phylogenetic trees of *Mortierella* spp. based on the ITS1-5.8S-ITS2 (ITS) region using RAxML version 8.1.5 software with the GTRGAMMAI model and bootstrapping (1000 replicates) with the rapid bootstrap analysis option. Bootstrap values >70% are shown at nodes. ML trees A, B, C, D, E, F and G (a single tree separately shown as Ga and Gb) were constructed for the phylogenetic groups 1, 2, 3, 4, 5, 6 and 7 of *Mortierella* spp. defined by Wagner *et al*. (2013), respectively and each alignment block used for phylogenetic analyses consisted of 342, 465, 421, 427, 427, 425 and 462 positions, respectively. Taxon names of isolates of *Mortierella* spp. examined for BRE were shown in bold and that of BRE-harbored isolates were shown in red. Abbreviations: T = ex-holotype; IT = ex-isotype; NT = ex-neotype; ST = ex-syntype; AUT = authentic strain. **M. sugadairana* and *M. oedorhiza* were newly reported species examined by isolates used in this study in Takashima *et al*. (2018) and Takashima *et al*. (submitted to Mycoscience), respectively.

Figure S2

LIVE/DEAD stained fluorescence images of BRE-detected isolates (A-F) and an isolate of *Mortierella* spp. (G), in which endofungal bacteria were not detected by the diagnostic PCR. Bright field images are shown beside each fluorescence image. Bacterial cells within hyphae and sporangiospores are indicated by arrowheads and arrows, respectively. Rod-shaped endofungal bacterial cells were detected within hypha of *M. elongata* FMR13-2 (A) and YTM18 (B), *M. alpina* YTM25 (C), *M. humilis* YTM36 (D), *M. verticillata* CBS 130.66 (E), and *M. basiparvispora* E1425 (F). Rod-shaped endofungal bacterial cells were also detected within sporangiospores of *M. alpina* YTM25 (C). No bacterial cells were detected within *M. basiparvispora* E1439 (G), which was identified as the same phylotype and isolated from the same location as *M. basiparvispora* E1425 (F). Scale bars: 10 μm.

References

Gardes, M., and T. D. Bruns. 1993. ITS primers with enhanced specificity for basidiomycetes-application to the identification of mycorrhizae and rusts. Mol. Ecol. 2:113-118.

Hijmans, R. J., S. E. Cameron, J. L. Parra, P. G. Jones and A. Jarvis. 2005. Very high resolution interpolated climate surfaces for global land areas. Int. J. Climatol. 25:1965-1978.

Lane, D.J. 1991. 16S/23S rRNA sequencing. In: Nucleic acid techniques in bacterial systematics. Stackebrandt, E., and Goodfellow, M., eds., John Wiley and Sons, New York, NY, pp. 115-175.

Takashima, Y., Y. Degawa, H. Ohta, and K. Narisawa. 2018. *Mortierella sugadairana*, a new homothallic species related to the firstly described heterothallic species in the genus. Mycoscience. 59:200-205.

Vilgalys, R., and M. Hester. 1990. Rapid genetic identification and mapping of enzymatically amplified ribosomal DNA from several *Cryptococcus* species. J. Bacteriol. 172:4238-4246.

Wagner, L., B. Stielow, K. Hoffmann, T. Petkovits, T. Papp, C. Vágvölgyi, G. S. de Hoog, G. Verkley, and K. Voigt. 2013. A comprehensive molecular phylogeny of the Mortierellales (Mortierellomycotina) based on nuclear ribosomal DNA. Persoonia. 30:77-93.

White, T. J., T. D. Bruns, S. B. Lee, and J. W. Taylor. 1990. Amplification and direct sequencing of fungal ribosomal RNA genes for phylogenetics. PCR protocols: a guide to methods and applications. 18:315-322.

Table S1 List of collection sites in this study and their estimated temperature data obtained from the WorldClim version 1.4.

Collection site no.	Putative climate*	Approximate geographic coordinate of collection site		Collection site name	Prefecture (in Japan)	Country	Annual mean temperature (°C)	Mean temperature of the warmest quarter (°C)	Mean temperature of the coldest quarter (°C)
		Latitude	Longitude						
1	Cool	43.69897	142.51032	Higashikawa-cho[a]	Hokkaido	Japan	6.0	19.0	-7.8
2	Cool	43.24480	143.55391	Ashoro-cho, Ashoro-gun[a]	Hokkaido	Japan	5.9	18.4	-7.8
3	Cool	43.07329	141.51096	Rakuno gakuen University, Ebetsu-shi	Hokkaido	Japan	7.6	19.7	-4.8
4	Cool	42.90562	143.18751	Midorigaoka Park, Obihiro-shi	Hokkaido	Japan	6.5	18.6	-6.7
5	Cool	37.21963	139.61718	Komado moor, Minamiaizu-cho	Fukushima	Japan	7.3	18.3	-3.5
6	Cool	36.05823	138.34598	Mt. Maruyama, Koumi-machi	Nagano	Japan	2.4	13.4	-8.5
7	Cool	36.52389	138.34747	Sugadaira research station, Mountain science center, University of Tsukuba, Ueda-shi	Nagano	Japan	6.3	17.4	-4.8
8	Cool	58.30153	-134.42201	Alaska[a]	-	USA	3.7	11.5	-4.1
9	Subtropical	26.85541	128.25092	Kayauchi Banta, Ginama, Kunigami-son, Kunigami-gun	Okinawa	Japan	21.8	27.5	15.9
10	Subtropical	26.65129	127.91995	Motobu-cho, Kunigami-gun	Okinawa	Japan	21.4	27.0	15.4
11	Subtropical	26.37704	126.75063	Nakandakari, Kumejima-cho, Kumejima-island	Okinawa	Japan	22.0	27.5	16.2
12	Subtropical	26.37693	126.77032	Around ruins of Uegusuku castle, Kumejima-cho, Kumejima-island	Okinawa	Japan	22.0	27.5	16.2
13	Subtropical	26.36090	126.76439	Darumayama Park, Kumejima-ch Kumejima-island	Okinawa	Japan	22.0	27.5	16.1
14	Subtropical	26.35506	126.74889	Uezu, Kumejima-island	Okinawa	Japan	22.4	27.9	16.5
15	Subtropical	26.31209	126.78965	Shimajiri, Kumejima-cho, Kumejima-island	Okinawa	Japan	22.2	27.6	16.3
16	Subtropical	26.29193	126.81247	Shimajirisaki, Kumejima-cho, Kumejima-island	Okinawa	Japan	22.5	27.9	16.7
17	Subtropical	26.22795	127.71512	Sueyoshi park, Shurisueyoshi-cho, Naha-shi	Okinawa	Japan	22.4	27.9	16.7
18	Subtropical	26.11947	127.67253	Itoman City central library, Itoman-shi	Okinawa	Japan	22.7	28.1	17.1

19	Subtropical	24.45532	122.95740	Mt. Kubura, Yonaguni-cho, Yonaguni island	Okinawa	Japan	23.4	27.9	18.3
20	Subtropical	24.42654	124.17987	Mt. Omotodake, Ishigaki-shi, Ishigaki-island	Okinawa	Japan	22.6	27.1	17.6
21	Temperate	38.03229	140.20390	Hinata cave, Takahata-machi	Yamagata	Japan	11.4	22.9	0.0
22	Temperate	37.85223	138.38964	Minamishinbo, Sado-shi, Sado isoland	Niigata	Japan	12.5	23.2	2.3
23	Temperate	37.66101	140.87740	Tateishi, Minamisouma-shi	Fukushima	Japan	11.8	22.2	1.9
24	Temperate	36.76774	140.69276	Hitana, Nakago-cho, Kitaibaraki-shi	Ibaraki	Japan	13.2	22.7	4.1
25	Temperate	36.19080	140.14929	Busshoji, Ishioka-shi	Ibaraki	Japan	12.8	23.0	2.7
26	Temperate	36.13435	140.13670	Shimo-ohshima, Tsukuba-shi	Ibaraki	Japan	13.6	23.8	3.5
27	Temperate	36.10882	140.10365	Around Institute of Art and Design Building in University of Tsukuba, Tsukuba-shi	Ibaraki	Japan	13.7	23.9	3.6
28	Temperate	36.09127	140.10957	Amakubo, Tsukuba-shi	Ibaraki	Japan	13.7	23.9	3.6
29	Temperate	36.03678	140.21510	Kakousou, Ibaraki Unversity, Ami-machi	Ibaraki	Japan	13.9	23.9	3.9
30	Temperate	36.03300	140.21192	Field Science Center, Ibaraki University College of Agriculture, Ami-machi	Ibaraki	Japan	13.9	23.9	3.9
31	Temperate	35.99487	140.24452	Mixed forest around Jindenike pond, Ami-machi	Ibaraki	Japan	14.0	24.0	4.1
32	Temperate	35.96902	140.63123	Kashima Shrine, Kashima-shi	Ibaraki	Japan	14.4	23.9	5.2
33	Temperate	35.36763	140.06488	Mariyatsu, Kisarazu-shi	Chiba	Japan	15.3	24.4	6.4
34	Temperate	35.04225	140.02033	Wada-machi, Minamiboso-shi[a]	Chiba	Japan	15.0	24.0	6.4
35	Temperate	35.70892	139.77422	Ueno, Taito-ku	Tokyo	Japan	15.5	25.2	6.0
36	Temperate	35.68649	139.74856	Fukiage Imperial Palace, Chiyoda-ku	Tokyo	Japan	15.5	25.2	6.0
37	Temperate	35.68140	139.76711	Tokyo[a]	Tokyo	Japan	15.7	25.3	6.2
38	Temperate	34.07577	139.47974	Izu, Miyake-mura, Miyakejima-island	Tokyo	Japan	16.5	24.2	8.9
39	Temperate	34.04715	139.50045	Around Mt. Nippana-Shinzan, Miyake-mura, Miyakejima-island	Tokyo	Japan	15.8	23.6	8.1

40	Temperate	37.51475	137.34445	Kongosaki, Suzu-shi	Ishikawa	Japan	13.1	23.5	3.3
41	Temperate	36.56421	136.65947	Kanazawa castle, Kanazawa-shi	Ishikawa	Japan	14.3	25.0	4.2
42	Temperate	36.39381	136.87815	Mt. Gokayama, Nanto-shi	Toyama	Japan	10.5	21.6	-0.6
43	Temperate	36.73062	139.59659	Mt. Mushinaki, Nikko-shi	Tochigi	Japan	9.6	20.0	-0.6
44	Temperate	36.38781	139.70265	Iwade-machi, Tochigi-shi	Tochigi	Japan	13.9	24.4	3.5
45	Temperate	36.41222	138.30763	Madake (Bamboo) forest in Tonoshiro, Ueda-shi	Nagano	Japan	10.9	22.1	-0.5
46	Temperate	36.40482	138.24469	Ueda Castle, Ueda-shi	Nagano	Japan	12.3	23.5	0.9
47	Temperate	36.39254	138.26241	Faculty of Textile Science and Technology, Shinshu University, Tokida, Ueda-shi	Nagano	Japan	12.0	23.3	0.6
48	Temperate	36.36864	138.25462	Mixed forest around Sugawa lake, Ueda-shi	Nagano	Japan	11.7	23.0	0.3
49	Temperate	36.26991	138.25823	Nagawa-machi, Chiisagata-gun[a]	Nagano	Japan	10.6	21.8	-0.7
50	Temperate	35.33010	139.49260	Shinbayashi Park, Fujisawa-shi	Kanagawa	Japan	15.5	24.9	6.4
51	Temperate	35.32902	139.54245	Kuzuharaoka shrine, Yamanouchi, Kamakura-shi	Kanagawa	Japan	15.6	24.9	6.5
52	Temperate	35.24191	139.12112	Iryuda, Odawara-shi	Kanagawa	Japan	13.8	23.3	4.5
53	Temperate	35.15847	139.13712	Manazuru-cho, Manazuru Peninsula	Kanagawa	Japan	15.6	24.8	6.9
54	Temperate	35.49398	138.67168	Cave around Mt. Fuji	Yamanashi	Japan	9.5	19.8	-0.8
55	Temperate	35.47493	138.66697	Around Narusawa ice cave, Narusawa-mura	Yamanashi	Japan	9.5	19.8	-0.8
56	Temperate	34.99482	138.52359	Miho-no-Matsubara, Shizuoka-shi	Shizuoka	Japan	16.2	25.4	7.2
57	Temperate	34.77523	138.01389	Around Kakegawa castle, Kakegawa-shi	Shizuoka	Japan	16.1	25.4	6.9
58	Temperate	35.24698	135.86841	Katsuragawabomura-cho, Ohtsu-shi	Shiga	Japan	11.3	22.0	0.8
59	Temperate	35.30955	135.71686	Ashiu Experimental Forest of Kyoto University, Miyamacho, Nantan-shi	Kyoto	Japan	11.6	22.3	1.1
60	Temperate	35.11321	135.81347	Ebumi shrine, Oharanomura-cho, Kyoto-shi	Kyoto	Japan	13.5	24.2	3.2

61	Temperate	34.47655	132.17092	Mominoki forest park, Hatsukaichi-shi	Hiroshima	Japan	9.9	20.3	-0.4
62	Temperate	33.02987	131.89428	Kariu cave, Kariu, Saiki	Oita	Japan	15.3	24.9	6.0
63	Temperate	31.37829	130.85208	Kanoya-shi[a]	Kagoshima	Japan	17.4	26.4	8.6
64	Temperate	53.56306	-3.07022	Lancashire, Freshfield[a]	-	UK	9.2	15.0	3.5
65	Temperate	53.23990	-2.69265	Cheshire, Delamere Forest	-	UK	9.0	14.6	3.6
66	Temperate	34.00071	-81.03481	South Carolina[a]	-	USA	17.2	26.2	7.6
67	Temperate	33.79484	-83.71323	Georgia, Monroe[a]	-	USA	16.0	24.9	6.5
68	Temperate	33.74900	-84.38798	Georgia[a]	-	USA	15.8	25.0	6.2

*Estimated temperature data of each collection site were obtained from the WorldClim version 1.4 at 2.5 minutes of a latitude/longitude degree spatial resolution data (Hijmans *et al*., 2005) and putative climates of each collection site were defined as follows in this study:
Cool; sites showing more than 10 °C and less than -3 °C in the mean temperature of the warmest and coldest quarter, respectively.
Subtropical; sites located in Ryukyu-islands in Japan with the latitude between 20° to 30° and showing more than 15 °C in the mean temperature of the coldest quarter.
Temperate; sites showing between -3 °C to 18 °C in the mean temperature of the coldest quarter except for sites defined as subtropical.
[a]Geographic coordinates were designated for the coodinate of a representative building such as a city hall in the state capital, city or town offices, and the Imperial Palace in Japan, and used in substitution for obtaining estimated temperature data for each collection site.

Table S2 List of isolates of *Mortierella* spp. used in this study.

Isolate no.	Other isolate name	Isolation date	Collection site no.	Putative climate	Isolation source	Type of substrate	Isolation method
YTM1	YD11-9	2011	7	Cool	Dung of rat	Dung	Direct plating
YTM3	OIG-10	14th May 2012	18	Subtropical	Soil under *Ficus microcarpa*	Soil	Direct plating
YTM4	2-4	16th July 2012	1	Cool	Root of eggplant	Plant	Surface washing
YTM5	OIG-2	14th May 2012	18	Subtropical	Soil under *Ficus microcarpa*	Soil	Direct plating
YTM6	Rh8	20th October 2013	29	Temperate	Soil under *Pteridium aquilinum*	Soil	Moist chamber method
YTM7	Rh5	20th October 2013	29	Temperate	Soil under *Pteridium aquilinum*	Soil	Moist chamber method
YTM8	OIG-4	14th May 2012	18	Subtropical	Soil under *Ficus microcarpa*	Soil	Direct plating
YTM9	KMK-7	14th May 2012	14	Subtropical	Soil	Soil	Direct plating
YTM10	33:113-1	20th October 2013	23	Temperate	Soil	Soil	Moist chamber method
YTM11	d_NN39:140	2nd November 2013	48	Temperate	Fecal pellets of centipede belong to the order *Lithobiomorpha*	Dung	Direct plating
YTM12	e_NN39:141	3rd November 2013	45	Temperate	Fecal pellets of centipede belong to the order *Lithobiomorpha*	Dung	Direct plating
YTM14	1-4	16th July 2012	1	Cool	Root of eggplant	Plant	Surface washing
YTM15	1-9	16th July 2012	1	Cool	Root of eggplant	Plant	Surface washing
YTM16	2-16	16th July 2012	1	Cool	Root of eggplant	Plant	Surface washing
YTM17	OHB2 30-13	14th May 2012	7	Cool	Soil under *Lonicera vidalii*	Soil	Direct plating
YTM18	OMG5	14th May 2012	10	Subtropical	Soil under *Tarenna gracilipes*	Soil	Direct plating
YTM19	OMG3	14th May 2012	10	Subtropical	Soil under *Tarenna gracilipes*	Soil	Direct plating
YTM20	ZDHs1-3	21th June 2014	31	Temperate	Root of *Huperzia serrata*	Plant	Surface washing
YTM21	OHB2 30-5	14th May 2012	7	Cool	Soil under *Lonicera vidalii*	Plant	Direct plating
YTM22	CLR1-1	October 2011	38	Temperate	Root of *Trachelospermum asiaticum*	Plant	Surface washing
YTM23	UM2	October 2011	38	Temperate	Root of *Arisaema urashima*	Plant	Surface washing
YTM24	40866	2013	7	Cool	Bone of bird on ground	Animal	Direct isolation
YTM25	UM5	October 2011	39	Temperate	Root of Pine	Plant	Surface washing
YTM26	UM1	October 2011	38	Temperate	Root of *Arisaema urashima*	Plant	Surface washing
YTM27	UM4	October 2011	38	Temperate	Root of *Trachelospermum asiaticum*	Plant	Surface washing
YTM28	b_NN39:150	11th November 2013	27	Temperate	Fecal pellets of centipede belong to the order *Scolopendromorpha*	Dung	Direct plating
YTM29	a_NN39:150	11th November 2013	27	Temperate	Fecal pellets of centipede belong to the order *Scolopendromorpha*	Dung	Direct plating
YTM30	Ir11-3	3rd June 2014	52	Temperate	Undescribed mucoromycotinan sporocarp	Fungi	Direct plating of sporocarp
YTM35	UM3	October 2011	38	Temperate	Stem of *Histiopteris incisa*	Plant	Surface washing
YTM36	NM-10	14th May 2012	43	Temperate	Soil under *Pinus thunbergii*	Plant	Direct plating

YTM37	ZDHs1-2	21th June 2014	31	Temperate	Root of *Huperzia serrata*	Plant	Surface washing
YTM38	SP4M	6th December 2014	7	Cool	Sporocarp of *Sphaerocreas pubescens*	Fungi	Direct plating of sporocarp
YTM39	IC-1, NBRC112366	20th June 2014	3	Cool	Root of tomato	Plant	Surface washing
YTM40	RK2-1	3rd July 2015	32	Temperate	Fruiting body of *Entoloma* sp.	Fungi	Direct plating of basidiocarp
YTM41	c_NN39:150	11th November 2013	27	Temperate	Fecal pellets of centipede belong to the order *Scolopendromorpha*	Dung	Direct plating
YTM42	LZ2	10th September 2014	58	Temperate	Root of *Lycopodium clavatum*	Plant	Surface washing
YTM43	LZ4	10th September 2014	58	Temperate	Root of *Lycopodium clavatum*	Plant	Surface washing
YTM44	LZ9	10th September 2014	60	Temperate	Root of *Huperzia serrata*	Plant	Surface washing
YTM45	LZ10	10th September 2014	60	Temperate	Root of *Huperzia serrata*	Plant	Surface washing
YTM46	SD2-3	29th September 2014	7	Cool	Soil under *Quercus crispula*	Plant	Baiting with toothpicks as a substrate
YTM47	MZA1-1	5th April 2015	53	Temperate	Soil	Soil	Moist chamber method
YTM48	MZA1-2	5th April 2015	53	Temperate	Soil	Soil	Moist chamber method
YTM49	MZA1-4	5th April 2015	53	Temperate	Soil	Soil	Moist chamber method
YTM50	MZA1-5	5th April 2015	53	Temperate	Soil	Soil	Moist chamber method
YTM51	MZA1-6	5th April 2015	53	Temperate	Soil	Soil	Moist chamber method
YTM52	MZA1-7	5th April 2015	53	Temperate	Soil	Soil	Moist chamber method
YTM53	MZB3-1	5th April 2015	53	Temperate	Soil under *Castanopsis sieboldii*	Soil	Moist chamber method
YTM54	MZB3-2	5th April 2015	53	Temperate	Soil under *Castanopsis sieboldii*	Soil	Moist chamber method
YTM55	MZB3-4	5th April 2015	53	Temperate	Soil under *Castanopsis sieboldii*	Soil	Moist chamber method
YTM56	MZDS1-1	21th April 2015	53	Temperate	Soil	Soil	Moist chamber method
YTM57	MZDS1-2	21th April 2015	53	Temperate	Soil	Soil	Moist chamber method
YTM58	MZDS1-3	21th April 2015	53	Temperate	Soil	Soil	Moist chamber method
YTM59	MZDS1-4	21th April 2015	53	Temperate	Soil	Soil	Moist chamber method
YTM60	NN39:220	2nd January 2013	44	Temperate	Fecal pellets of cave weta belong to the order *Rhaphidophoridae*	Dung	Direct plating
YTM61	A_NN41:131	30th May 2015	15	Subtropical	Forest soil	Soil	Moist chamber method
YTM62	NN41:160	10th June 2015	35	Temperate	Dung of rat	Dung	Direct plating of dung
YTM63	NN41:161	10th June 2015	35	Temperate	Dung of rat	Dung	Direct plating of dung
YTM64	Kume_NN41:122	22th May 2015	17	Subtropical	Fecal pellets of centipede belong to the order *Scolopendromorpha*	Dung	Direct isolation
YTM65	12DS11_NN36:262_277	2nd March 2012	51	Temperate	Decayed wood	Plant	Moist chamber method
YTM66	Seto	15th June 2015	7	Cool	Fruiting body of *Lycogala epidendrum*	Animal	Direct isolation
YTM68	Fuji NN37:238	December 2013	54	Temperate	A dead body of bat	Animal	Direct isolation
YTM69	cpt a4a NN39:249, 275	19th January 2013	47	Temperate	Dried soil	Soil	Moist chamber method
YTM70	cpt a2b NN39:249, 275	19th January 2013	47	Temperate	Soil under Juniper plant	Soil	Moist chamber method
YTM71	cpt a5a NN39:249, 275	19th January 2013	47	Temperate	Soil	Soil	Moist chamber method

YTM72	cpt b4b NN39:249, 275	19th January 2013	46	Temperate	Dead body of *Armadillidium vulgare* placed on dried soil in the interspace of stone wall	Animal	Moist chamber method
YTM73	Hyuga_NN39:247	18th January 2013	21	Temperate	On the surface of a millipede	Animal	Plating by walking of a millipede on water agar
YTM74	Oki1_NN39:113	14th October 2013	9	Subtropical	Soil around the field where Isopoda were numerous	Soil	Moist chamber method
YTM75	Oki2_NN39:113	14th October 2013	9	Subtropical	Soil around the field where Isopoda were numerous	Soil	Moist chamber method
YTM76	Oki3_NN39:113	14th October 2013	9	Subtropical	Soil around the field where Isopoda were numerous	Soil	Moist chamber method
YTM78	Mh12	2nd July 2015	25	Temperate	Root of *Monotropastrum humile*	Plant	Surface washing
YTM79	Mh16	2nd July 2015	25	Temperate	Root of *Monotropastrum humile*	Plant	Surface washing
YTM80	Mh18	2nd July 2015	25	Temperate	Root of *Monotropastrum humile*	Plant	Surface washing
YTM81	Mh22	2nd July 2015	25	Temperate	Root of *Monotropastrum humile*	Plant	Surface washing
YTM82	Mh28	2nd July 2015	25	Temperate	Root of *Monotropastrum humile*	Plant	Surface washing
YTM83	Mh31	2nd July 2015	25	Temperate	Root of *Monotropastrum humile*	Plant	Surface washing
YTM84	Mh33	2nd July 2015	25	Temperate	Root of *Monotropastrum humile*	Plant	Surface washing
YTM85	Mh34	2nd July 2015	25	Temperate	Root of *Monotropastrum humile*	Plant	Surface washing
YTM86	Mh38	2nd July 2015	25	Temperate	Root of *Monotropastrum humile*	Plant	Surface washing
YTM87	Mh40	2nd July 2015	25	Temperate	Root of *Monotropastrum humile*	Plant	Surface washing
YTM88	Mh41	2nd July 2015	25	Temperate	Root of *Monotropastrum humile*	Plant	Surface washing
YTM89	Mh42	2nd July 2015	25	Temperate	Root of *Monotropastrum humile*	Plant	Surface washing
YTM90	MEiA3-1	30th September 2015	6	Cool	Sporocarp of *Endogone incrassata*	Fungi	Direct plating of sporocarp
YTM91	MEiA3-2	30th September 2015	6	Cool	Sporocarp of *Endogone incrassata*	Fungi	Direct plating of sporocarp
YTM92	MEiA4-1	30th September 2015	6	Cool	Sporocarp of *Endogone incrassata*	Fungi	Direct plating of sporocarp
YTM93	MEiA24-1	30th September 2015	6	Cool	Sporocarp of *Endogone incrassata*	Fungi	Direct plating of sporocarp
YTM94	MEiA27-1	30th September 2015	6	Cool	Sporocarp of *Endogone incrassata*	Fungi	Direct plating of sporocarp
YTM95	MEiA27-2	30th September 2015	6	Cool	Sporocarp of *Endogone incrassata*	Fungi	Direct plating of sporocarp
YTM96	IK1	27th October 2015	26	Temperate	Soil around the field where *Armadillidium vulgare* were numerous	Soil	Moist chamber method
YTM97	IK2	27th October 2015	26	Temperate	Soil around the field where *Armadillidium vulgare* were numerous	Soil	Moist chamber method
YTM98	Iky2	27th October 2015	26	Temperate	Fallen straws from a thatched roof	Plant	Direct plating
YTM99	Iky3	27th October 2015	26	Temperate	Fallen straws from a thatched roof	Plant	Direct plating
YTM100	KA2	30th October 2015	15	Subtropical	Forest soil	Soil	Moist chamber method
YTM104	KC2	28th October 2015	16	Subtropical	Soil under *Pandanus odoratissimus*	Soil	Moist chamber method
YTM108	KC6	28th October 2015	16	Subtropical	Soil under *Pandanus odoratissimus*	Soil	Moist chamber method

YTM110	KD1	28th October 2015	13	Subtropical	Soil under *Pinus luchuensis*	Soil	Moist chamber method
YTM111	KD2	28th October 2015	13	Subtropical	Soil under *Pinus luchuensis*	Soil	Moist chamber method
YTM112	KD3	28th October 2015	13	Subtropical	Soil under *Pinus luchuensis*	Soil	Moist chamber method
YTM113	KD5	28th October 2015	13	Subtropical	Soil under *Pinus luchuensis*	Soil	Moist chamber method
YTM115	KE2	27th October 2015	12	Subtropical	Lawn field soil	Soil	Moist chamber method
YTM117	KG3	27th October 2015	11	Subtropical	Crop land soil	Soil	Moist chamber method
YTM119	KG8	30th October 2015	11	Subtropical	Crop land soil	Soil	Moist chamber method
YTM120	KG9	30th October 2015	11	Subtropical	Crop land soil	Soil	Moist chamber method
YTM122	NA1	27th October 2015	40	Temperate	Forest soil	Soil	Moist chamber method
YTM123	OM1-1	5th November 2015	4	Cool	Soil under *Abies veitchii*	Soil	Moist chamber method
YTM124	OM1-2	5th November 2015	4	Cool	Soil under *Abies veitchii*	Soil	Moist chamber method
YTM125	OM1-3	5th November 2015	4	Cool	Soil under *Abies veitchii*	Soil	Moist chamber method
YTM126	OM1-4	5th November 2015	4	Cool	Soil under *Abies veitchii*	Soil	Moist chamber method
YTM127	OM1-6	5th November 2015	4	Cool	Soil under *Abies veitchii*	Soil	Moist chamber method
YTM128	OM1-7-3	5th November 2015	4	Cool	Soil under *Abies veitchii*	Soil	Moist chamber method
YTM129	OM2-4	5th November 2015	4	Cool	Soil under *Pinus densiflora*	Soil	Moist chamber method
YTM130	OM2-5	5th November 2015	4	Cool	Soil under *Pinus densiflora*	Soil	Moist chamber method
YTM131	OM2-9	5th November 2015	4	Cool	Soil under *Pinus densiflora*	Soil	Moist chamber method
YTM132	OM2-10	5th November 2015	4	Cool	Soil under *Pinus densiflora*	Soil	Moist chamber method
YTM133	OM4-1	5th November 2015	4	Cool	Soil around the field where *Ligidium japonicum* were numerous	Soil	Moist chamber method
YTM134	OM4-2	5th November 2015	4	Cool	Soil around the field where *Ligidium japonicum* were numerous	Soil	Moist chamber method
YTM135	OM4-5	5th November 2015	4	Cool	Soil around the field where *Ligidium japonicum* were numerous	Soil	Moist chamber method
YTM137	OM5-2	7th November 2015	4	Cool	Muck soil	Soil	Moist chamber method
YTM138	OM5-4	7th November 2015	4	Cool	Muck soil	Soil	Moist chamber method
YTM139	OM5-5	7th November 2015	4	Cool	Muck soil	Soil	Moist chamber method
YTM140	IKMc1	12th December 2015	26	Temperate	A dead body of *Armadillidium vulgare*	Animal	Moist chamber method
YTM141	IKMc2	12th December 2015	26	Temperate	A dead body of *Armadillidium vulgare*	Animal	Moist chamber method
YTM142	IKMc3	12th December 2015	26	Temperate	A dead body of *Armadillidium vulgare*	Animal	Moist chamber method
YTM143	IKMc4	12th December 2015	26	Temperate	A dead body of *Armadillidium vulgare*	Animal	Moist chamber method
YTM144	IKMcs1	12th December 2015	26	Temperate	A dead body of *Armadillidium vulgare*	Animal	Moist chamber method
YTM145	IKMcs2	12th December 2015	26	Temperate	A dead body of *Armadillidium vulgare*	Animal	Moist chamber method
YTM146	KA5-1	12th December 2015	15	Subtropical	Forest soil	Soil	Moist chamber method
YTM147	KA5-2	12th December 2015	15	Subtropical	Forest soil	Soil	Moist chamber method
YTM148	KA5-3	12th December 2015	15	Subtropical	Forest soil	Soil	Moist chamber method
YTM149	KA5-4	12th December 2015	15	Subtropical	Forest soil	Soil	Moist chamber method

YTM150	OM3-8M	12th December 2015	4	Cool	Soil under *Betula platyphylla*	Soil	Moist chamber method
YTM151	OM4-7M	12th December 2015	4	Cool	Soil around the field where *Ligidium japonicum* were numerous	Soil	Moist chamber method
YTM152	OM4-6	12th December 2015	4	Cool	Soil around the field where *Ligidium japonicum* were numerous	Soil	Moist chamber method
YTM153	KA5M	12th December 2015	15	Subtropical	Forest soil	Soil	Moist chamber method
YTM154	KA6M	12th December 2015	15	Subtropical	Forest soil	Soil	Moist chamber method
YTM155	KA7M	12th December 2015	15	Subtropical	Forest soil	Soil	Moist chamber method
YTM156	KG4	27th October 2015	11	Subtropical	Crop land Soil	Soil	Moist chamber method
YTM157	KG10	30th October 2015	11	Subtropical	Crop land Soil	Soil	Moist chamber method
YTM158	OM2-11	5th November 2015	4	Cool	Soil under under *Pinus densiflora*	Soil	Moist chamber method
YTM159	NN42:20-1	3rd February 2016	37	Temperate	Fruiting body of *Fuligo septica*	Animal	Direct isolation
YTM160	Goka2-1	9th February 2016	42	Temperate	Soil	Soil	Direct plating
YTM161	Goka2-3	9th February 2016	42	Temperate	Soil	Soil	Direct plating
YTM162	Kana6-2	9th February 2016	42	Temperate	Soil	Soil	Direct plating
YTM163	Kana6-3	9th February 2016	41	Temperate	Soil	Soil	Direct plating
YTM164	YN1-1	13th February 2016	19	Subtropical	Forest soil	Soil	Moist chamber method
YTM165	YN1-2	13th February 2016	19	Subtropical	Forest soil	Soil	Moist chamber method
YTM166	YN2-1	13th February 2016	19	Subtropical	Forest soil	Soil	Moist chamber method
YTM167	YN2-3	13th February 2016	19	Subtropical	Forest soil	Soil	Moist chamber method
YTM168	YNS2-1	13th February 2016	19	Subtropical	Forest soil	Soil	Moist chamber method
YTM169	YN2-2	13th February 2016	19	Subtropical	Forest soil	Soil	Moist chamber method
YTM170	PYN1-1	16th February 2016	19	Subtropical	Forest soil	Soil	Direct plating
YTM171	PYN1-2	16th February 2016	19	Subtropical	Forest soil	Soil	Direct plating
YTM172	PYN1-3	16th February 2016	19	Subtropical	Forest soil	Soil	Direct plating
YTM173	PYN1-4	16th February 2016	19	Subtropical	Forest soil	Soil	Direct plating
YTM174	PYN1-5	16th February 2016	19	Subtropical	Forest soil	Soil	Direct plating
YTM175	PYN1-6	16th February 2016	19	Subtropical	Forest soil	Soil	Direct plating
YTM176	PYN2-1	16th February 2016	19	Subtropical	Forest soil	Soil	Direct plating
YTM177	PYN2-2	16th February 2016	19	Subtropical	Forest soil	Soil	Direct plating
YTM178	PYN5-2	16th February 2016	20	Subtropical	Forest soil	Soil	Direct plating
YTM179	PNIC1	16th February 2016	55	Temperate	Soil under *Picea torano*	Soil	Direct plating
YTM180	PNIC2	16th February 2016	55	Temperate	Soil under *Picea torano*	Soil	Direct plating
YTM181	PNIC3	16th February 2016	55	Temperate	Soil under *Picea torano*	Soil	Direct plating
YTM182	PNIC4	16th February 2016	55	Temperate	Soil under *Picea torano*	Soil	Direct plating
YTM183	PNIC5	16th February 2016	55	Temperate	Soil under *Picea torano*	Soil	Direct plating
YTM184	PNIC6	16th February 2016	55	Temperate	Soil under *Picea torano*	Soil	Direct plating
YTM185	PNIC7	16th February 2016	55	Temperate	Soil under *Picea torano*	Soil	Direct plating

YTM186	PNIC8	16th February 2016	55	Temperate	Soil under *Picea torano*	Soil	Direct plating
YTM187	PNIC9	16th February 2016	55	Temperate	Soil under *Picea torano*	Soil	Direct plating
YTM188	SMM1	13th February 2016	56	Temperate	Soil under under *Pinus densiflora*	Soil	Moist chamber method
YTM189	SMM2	13th February 2016	56	Temperate	Soil under under *Pinus densiflora*	Soil	Moist chamber method
YTM190	PSMM1	16th February 2016	56	Temperate	Soil under under *Pinus densiflora*	Soil	Direct plating
YTM191	PSMM2	16th February 2016	56	Temperate	Soil under under *Pinus densiflora*	Soil	Direct plating
YTM192	SMMS1	13th February 2016	56	Temperate	Soil under under *Pinus densiflora*	Soil	Moist chamber method
YTM193	SMMS2	13th February 2016	56	Temperate	Soil under under *Pinus densiflora*	Soil	Moist chamber method
YTM194	SMMS3	13th February 2016	56	Temperate	Soil under under *Pinus densiflora*	Soil	Moist chamber method
YTM195	SMMS4	13th February 2016	56	Temperate	Soil under under *Pinus densiflora*	Soil	Moist chamber method
YTM196	PSKC1-1	16th February 2016	57	Temperate	Sand in the interspace of stone wall	Soil	Direct plating
YTM197	PSKC1-2	16th February 2016	57	Temperate	Sand in the interspace of stone wall	Soil	Direct plating
YTM198	PSKC1-3	16th February 2016	57	Temperate	Sand in the interspace of stone wall	Soil	Direct plating
YTM199	PSKC1-4	16th February 2016	57	Temperate	Sand in the interspace of stone wall	Soil	Direct plating
YTM200	PSKC1-5	16th February 2016	57	Temperate	Sand in the interspace of stone wall	Soil	Direct plating
YTM201	PSKC1-6	16th February 2016	57	Temperate	Sand in the interspace of stone wall	Soil	Direct plating
YTM202	PSKC2-1	16th February 2016	57	Temperate	Sand in the interspace of stone wall	Soil	Direct plating
YTM203	PSKC2-2	16th February 2016	57	Temperate	Sand in the interspace of stone wall	Soil	Direct plating
YTM204	PSKC2-3	16th February 2016	57	Temperate	Sand in the interspace of stone wall	Soil	Direct plating
YTM205	SADO-Sh-Rami1	12th May 2016	22	Temperate	Soil	Soil	Moist chamber method
YTM206	NAKA1-Mor3	12th May 2016	24	Temperate	Soil	Soil	Direct plating
YTM207	NAKA2-Mor1	12th May 2016	24	Temperate	Soil	Soil	Direct plating
YTM208	NAKA2-Mor3	12th May 2016	24	Temperate	Soil	Soil	Direct plating
YTM209	NAKA-Sh-Mor1	12th May 2016	24	Temperate	Soil	Soil	Moist chamber method
YTM210	NAKA-Sh-Mor2	12th May 2016	24	Temperate	Soil	Soil	Moist chamber method
YTM211	NAKA-Sh-Z	12th May 2016	24	Temperate	Soil	Soil	Moist chamber method
YTM212	SADO-Sh-Mor1	12th May 2016	22	Temperate	Soil	Soil	Moist chamber method
YTM213	SADO-Sh-Mor2	12th May 2016	22	Temperate	Soil	Soil	Moist chamber method
YTM214	SADO-Sh-Mor4	12th May 2016	22	Temperate	Soil	Soil	Moist chamber method
YTM215	SADO-S2-Mor2	12th May 2016	22	Temperate	Soil	Soil	Direct plating
YTM217	SADO-Sh-Mor3	12th May 2016	22	Temperate	Soil	Soil	Moist chamber method
YTM218	SADO-S2-Mor1	12th May 2016	22	Temperate	Soil	Soil	Direct plating
YTM219	SADO-S2-Mor3	12th May 2016	22	Temperate	Soil	Soil	Direct plating
YTM220	OB1_N_1B	7th November 2016	5	Cool	Root of *Fagus crenata*	Plant	Surface washing
YTM222	OB2_N_1B	7th November 2016	5	Cool	Root of *Fagus crenata*	Plant	Surface washing
YTM223	OB2_NS_2A	7th November 2016	5	Cool	Root of *Fagus crenata*	Plant	Surface washing and sterilization with calcium hypochlorite

YTM224	OB2_NS_2B	7th November 2016	5	Cool	Root of *Fagus crenata*	Plant	Surface washing and sterilization with calcium hypochlorite
YTM225	OE1_N_1A	3rd November 2016	5	Cool	Root of *Fagus crenata*	Plant	Surface washing
YTM226	OE1_N_2A	3rd November 2016	5	Cool	Root of *Fagus crenata*	Plant	Surface washing
E1425	-	1st August 2015	61	Temperate	Soil	Soil	Dilution plate method
E1439	-	1st August 2015	61	Temperate	Soil	Soil	Dilution plate method
E1600	-	1st August 2015	61	Temperate	Soil	Soil	Dilution plate method
E1931	-	13th August 2015	63	Temperate	Soil	Soil	Moist chamber method
E2010s1	-	1st August 2015	61	Temperate	Soil	Soil	Moist chamber method
E2092	-	12th August 2015	49	Temperate	Litter	Soil	Moist chamber method
E2642	-	22th September 2015	2	Cool	Soil	Soil	Moist chamber method
FMR13-2	-	-	30	Temperate	Soil	Soil	Direct plating
FMR23-1	-	-	30	Temperate	Soil	Soil	Direct plating
FMR23-6	-	-	30	Temperate	Soil	Soil	Direct plating
FMR23-9	-	-	30	Temperate	Soil	Soil	Direct plating
NBRC8569	-	-	-	Unknown	-	-	-
NBRC8574	CBS 345.66	-	8	Cool	-	-	-
NBRC32831	CBS287.96	2nd August 1995	28	Temperate	Soil under *Quercus mirsinaefolia* forest	Soil	Moist chamber method
NBRC32834	CBS288.96	2nd August 1995	28	Temperate	Soil under *Quercus mirsinaefolia* forest	Soil	Moist chamber method
NBRC100829	CBS116202	2004	62	Temperate	Dung of bat, *Rhinolophus* sp.	Dung	-
NBRC104553	-	-	7	Cool	Decayed twig, *Fagus crenata*	Plant	-
NBRC104986	-	2006	59	Temperate	Beech log	Plant	-
NBRC112302	-	2014	34	Temperate	Compost	Plant	-
NBRC112303	-	2015	33	Temperate	Compost	Plant	-
NBRC109920	-	30th July 2013	36	Temperate	Fecal pellets of the centipede *Thereuopoda clunifera*	Dung	Direct plating
NBRC109921	-	24th April 2013	50	Temperate	Fecal pellets of the centipede *Thereuopoda clunifera*	Dung	Direct plating
CBS121.71	-	-	67	Temperate	Soil under golf turf-grass	Soil	-
CBS122.71	-	-	67	Temperate	Soil under golf turf-grass	Soil	-
CBS130.66	-	-	64	Temperate	Sandy forest soil	Soil	-
CBS131.66	-	-	64	Temperate	Sandy forest soil	Soil	-
CBS315.61	-	-	65	Temperate	Soil, iron-humus podzol	Soil	-
CBS316.61	-	-	65	Temperate	Soil, iron-humus podzol	Soil	-
CBS277.71	-	-	68	Temperate	Forest soil	Soil	-
CBS278.71	-	-	68	Temperate	Forest soil	Soil	-
CBS222.35	-	-	-	Unknown	Soil from Pinus forest	Soil	-

CBS443.68	-	-	66	Temperate	Pinus bark of stump	Plant	-
CBS857.70	-	-	-	Unknown	Decaying needle, *Pinus sylvestris*	Plant	-
CBS858.70	-	-	-	Unknown	Decaying root, *Dactylis glomerata*	Plant	-

Table S3 List of primers used in this study.

Primer name	Target organism	Target site	Primer sequence	Primer information
10F	Bacteria	16S ribosomal RNA gene	AGTTTGATATCCTGGCTCAG	Binding positions 10–27 of the *Escherichia coli* 16S rRNA gene
27F	Bacteria	16S ribosomal RNA gene	AGAGTTTGATCMTGGCTCAG	Lane, 1991
800F	Bacteria	16S ribosomal RNA gene	ATTAGATACCCTGGTA	Binding positions 800-815 of the *E. coli* 16S rRNA gene
786R	Bacteria	16S ribosomal RNA gene	GACTACCAGGGTATCTAATC	Binding positions 805-786 of the *E. coli* 16S rRNA gene
926R	Bacteria	16S ribosomal RNA gene	CCGTCAATTCMTTTRAGTTT	Lane, 1991
1492R	Bacteria	16S ribosomal RNA gene	CGGTTACCTTGTTACGACTT	Lane, 1991
1541R	Bacteria	16S ribosomal RNA gene	AAGGAGGTGATCCAGCCG	Binding positions 1541-1524 of the *E. coli* 16S rRNA gene
ITS1F	Fungi	Internal transcribed spacer regions	CTTGGTCATTTAGAGGAAGTAA	Gardes and Bruns, 1993
ITS3	Fungi	Internal transcribed spacer regions	GCATCGATGAAGAACGCAGC	White *et al*., 1990
ITS4	Fungi	Internal transcribed spacer regions	TCCTCCGCTTATTGATATGC	White *et al*., 1990
ITS5	Fungi	Internal transcribed spacer regions	GGAAGTAAAAGTCGTAACAAGG	White *et al*., 1990
LR0R	Fungi	28S ribosomal RNA gene	ACCCGCTGAACTTAAGC	Vilgalys unpublished (http://www.botany.duke.edu/fungi/mycolab)
LR5	Fungi	28S ribosomal RNA gene	TCCTGAGGGAAACTTCG	Vilgalys and Hester, 1990

Table S4 Phylogenetically identified species of isolates used in this study based on blastn searches and phylogenetic analyses using ITS sequences.

Isolate no.	Phylogenetic group sense Wagner *et al*. (2013)	Species no.	Species identified by integrating blastn searches and phylogenetic analyses[†]	Putative species assigned by phylogenetic analyses	Top match species matched by blastn searches	Cultutre collection no. of top match phylotypes	Accession no. of top match phylotypes	Query accssion no.	Query length	Evalue	Identity (%)
YTM92	Group 1	1	*Mortierella alliacea*	*Mortierella alliacea*	*Mortierella alliacea*	CBS 894.68	JX975990	MF423575	552	0	100.0
E1931	Group 1	2	*Mortierella chienii*	*Mortierella chienii*	*Mortierella chienii*	CBS 289.96	JX975898	MF423488	573	0	99.5
NBRC32831	Group 1	2	*Mortierella chienii*	*Mortierella chienii*	*Mortierella chienii*	CBS 287.96	JX976013	MH802519	571	0	100.0
NBRC32834	Group 1	2	*Mortierella chienii*	*Mortierella chienii*	*Mortierella chienii*	CBS 287.96	JX976013	MF403052	571	0	98.9
YTM47	Group 1	2	*Mortierella chienii*	*Mortierella chienii*	*Mortierella chienii*	CBS 289.96	JX975898	MF423532	572	0	98.3
YTM48	Group 1	2	*Mortierella chienii*	*Mortierella chienii*	*Mortierella chienii*	CBS 287.96	JX976013	MF423533	593	0	98.8
YTM51	Group 1	2	*Mortierella chienii*	*Mortierella chienii*	*Mortierella chienii*	CBS 289.96	JX975898	MF423536	573	0	99.8
YTM52	Group 1	2	*Mortierella chienii*	*Mortierella chienii*	*Mortierella chienii*	CBS 287.96	JX976013	MF423537	597	0	99.8
YTM56	Group 1	2	*Mortierella chienii*	*Mortierella chienii*	*Mortierella chienii*	CBS 287.96	JX976013	MF423541	533	0	98.7
YTM57	Group 1	2	*Mortierella chienii*	*Mortierella chienii*	*Mortierella chienii*	CBS 289.96	JX975898	MF423542	535	0	100.0
YTM58	Group 1	2	*Mortierella chienii*	*Mortierella chienii*	*Mortierella chienii*	CBS 289.96	JX975898	MF423543	535	0	100.0
YTM59	Group 1	2	*Mortierella chienii*	*Mortierella chienii*	*Mortierella chienii*	CBS 289.96	JX975898	MF423544	535	0	100.0
YTM61	Group 1	2	*Mortierella chienii*	*Mortierella chienii*	*Mortierella chienii*	CBS 287.96	JX976013	MF423546	571	0	98.6
E1600	Group 1	3	*Mortierella cystojenkinii*	*Mortierella cystojenkinii*	*Mortierella cystojenkinii*	CBS 456.71	JX976030	MF423487	538	0	99.8
YTM94	Group 1	4	*Mortierella pulchella*	*Mortierella pulchella*	*Mortierella pulchella*	CBS 675.88	JX976082	MF423577	550	0	100.0
YTM95	Group 1	4	*Mortierella pulchella*	*Mortierella pulchella*	*Mortierella pulchella*	CBS 675.88	JX976082	MF423578	545	0	100.0
YTM119	Group 1	5	*Mortierella selenospora*	*Mortierella selenospora*	*Mortierella selenospora*	CBS 811.68	JX975875	MF423592	636	0	97.5
YTM157	Group 1	5	*Mortierella selenospora*	*Mortierella selenospora*	*Mortierella selenospora*	CBS 811.68	JX975875	MF423628	635	0	97.5
YTM223	Group 1	6	*Mortierella sossauensis*	*Mortierella sossauensis*	*Mortierella sossauensis*	CBS 890.72	JX975865	MF423692	508	0	99.8
YTM224	Group 1	6	*Mortierella sossauensis*	*Mortierella sossauensis*	*Mortierella sossauensis*	CBS 890.72	JX975865	MF423693	544	0	100.0
E1425	Group 1	7	*Mortierella basiparvispora*	"parvispora-jenkinii complex"	*Mortierella basiparvispora*	CBS 517.72	JX976048	MF423485	530	0	96.2
E1439	Group 1	7	*Mortierella basiparvispora*	"parvispora-jenkinii complex"	*Mortierella basiparvispora*	CBS 517.72	JX976048	MF423486	530	0	96.2
E2642	Group 1	7	*Mortierella basiparvispora*	"parvispora-jenkinii complex"	*Mortierella basiparvispora*	CBS 517.72	JX976048	MF423491	533	0	96.4
YTM11	Group 1	8	*Mortierella jenkinii*	"parvispora-jenkinii complex"	*Mortierella jenkinii*	CBS 667.70	JX976088	MF423501	518	0	98.1
CBS315.61	Group 1	9	*Mortierella parvispora*	"parvispora-jenkinii complex"	*Mortierella parvispora*	CBS 315.61	JX976104	JX976104	-	-	-
CBS316.61	Group 1	9	*Mortierella parvispora*	"parvispora-jenkinii complex"	*Mortierella parvispora*	CBS 316.61	JX976029	JX976029	-	-	-
E2010s1	Group 1	9	*Mortierella parvispora*	"parvispora-jenkinii complex"	*Mortierella parvispora*	CBS 315.61	JX976104	MF423489	554	0	93.2
E2092	Group 1	9	*Mortierella parvispora*	"parvispora-jenkinii complex"	*Mortierella parvispora*	FSU 10712	JX975941	MF423490	531	0	99.2
NBRC104986	Group 1	9	*Mortierella parvispora*	"parvispora-jenkinii complex"	*Mortierella parvispora*	CBS 445.68	JX976049	MH802522	512	0	98.2
NBRC8574	Group 1	9	*Mortierella parvispora*	"parvispora-jenkinii complex"	*Mortierella parvispora*	FSU 10712	JX975941	MH802517	508	0	99.4
YTM66	Group 1	9	*Mortierella parvispora*	"parvispora-jenkinii complex"	*Mortierella parvispora*	CBS 445.68	JX976049	MF423551	514	0	97.0
YTM125	Group 1	9	*Mortierella parvispora*	"parvispora-jenkinii complex"	*Mortierella parvispora*	FSU 10712	JX975941	MF423597	541	0	99.8
YTM126	Group 1	9	*Mortierella parvispora*	"parvispora-jenkinii complex"	*Mortierella parvispora*	FSU 10712	JX975941	MF423598	537	0	99.8
YTM130	Group 1	9	*Mortierella parvispora*	"parvispora-jenkinii complex"	*Mortierella parvispora*	FSU 10712	JX975941	MF423602	537	0	99.6
NBRC104553	Group 1	10	*Mortierella sugadairana* *	*Mortierella sugadairana* *	*Mortierella parvispora*	FSU 10712	JX975941	MF510830	507	0	99.8
YTM39	Group 1	10	*Mortierella sugadairana* *	*Mortierella sugadairana* *	*Mortierella parvispora*	FSU 10789	JX976065	MF423524	510	0	99.8
YTM128	Group 1	10	*Mortierella sugadairana* *	*Mortierella sugadairana* *	*Mortierella parvispora*	FSU 10712	JX975941	MF423600	537	0	99.8
YTM90	Group 1	11	*Mortierella* sp. 1	*Mortierella* sp. 1	*Mortierella alliacea*	CBS 106.78	JX976019	MF423573	547	0	97.4
YTM91	Group 1	11	*Mortierella* sp. 1	*Mortierella* sp. 1	*Mortierella alliacea*	CBS 106.78	JX976019	MF423574	548	0	97.5
YTM133	Group 1	12	*Mortierella* sp. 2	*Mortierella* sp. 2	*Mortierella jenkinii*	CBS 667.70	JX976088	MF423605	551	0	93.9
YTM166	Group 1	13	*Mortierella* sp. 3	*Mortierella* sp. 3	*Mortierella dichotoma*	CBS 221.35	JX975842	MF423637	542	0	95.2
YTM14	Group 2	14	*Mortierella clonocystis*	*Mortierella clonocystis*	*Mortierella* sp. "minutissima"	CBS 226.35	JX976092	MF423503	527	0	100.0
YTM15	Group 2	14	*Mortierella clonocystis*	*Mortierella clonocystis*	*Mortierella* sp. "minutissima"	CBS 226.35	JX976092	MF423504	557	0	100.0
YTM16	Group 2	14	*Mortierella clonocystis*	*Mortierella clonocystis*	*Mortierella* sp. "minutissima"	CBS 226.35	JX976092	MF423505	559	0	100.0
YTM175	Group 2	15	*Mortierella epicladia*	*Mortierella epicladia*	*Mortierella epicladia*	CBS 355.76	JX976130	MF423646	546	0	100.0
CBS222.35	Group 2	16	*Mortierella humilis*	"humilis-verticillata complex"	*Mortierella humilis*	CBS 222.35	HQ630325	HQ630325	-	-	-

CBS443.68	Group 2	16	*Mortierella humilis*	"humilis-verticillata complex"	*Mortierella humilis*	CBS 443.68	JX976097	JX976097	-	-	-
YTM36	Group 2	16	*Mortierella humilis*	"humilis-verticillata complex"	*Mortierella humilis*	CBS 180.72	JX976125	MF423521	557	0	100.0
YTM46	Group 2	16	*Mortierella humilis*	"humilis-verticillata complex"	*Mortierella humilis*	CBS 180.72	JX976125	MF423531	521	0	100.0
YTM187	Group 2	16	*Mortierella humilis*	"humilis-verticillata complex"	*Mortierella humilis*	CBS 180.72	JX976125	MF423658	521	0	100.0
YTM208	Group 2	16	*Mortierella humilis*	"humilis-verticillata complex"	*Mortierella humilis*	CBS 222.35	HQ630325	MF423679	582	0	100.0
YTM211	Group 2	16	*Mortierella humilis*	"humilis-verticillata complex"	*Mortierella humilis*	CBS 180.72	JX976125	MF423682	521	0	100.0
YTM213	Group 2	16	*Mortierella humilis*	"humilis-verticillata complex"	*Mortierella humilis*	CBS 180.72	JX976125	MF423684	519	0	100.0
YTM220	Group 2	16	*Mortierella humilis*	"humilis-verticillata complex"	*Mortierella humilis*	CBS 180.72	JX976125	MF423690	498	0	100.0
YTM225	Group 2	16	*Mortierella humilis*	"humilis-verticillata complex"	*Mortierella humilis*	CBS 180.72	JX976125	MF423694	521	0	100.0
CBS130.66	Group 2	17	*Mortierella verticillata*	"humilis-verticillata complex"	*Mortierella verticillata*	CBS 130.66	JX976007	JX976007	-	-	-
CBS131.66	Group 2	17	*Mortierella verticillata*	"humilis-verticillata complex"	*Mortierella verticillata*	CBS 131.66	JX975886	JX975886	-	-	-
YTM35	Group 2	17	*Mortierella verticillata*	"humilis-verticillata complex"	*Mortierella verticillata*	CBS 315.52	JX975943	MF423520	551	0	100.0
YTM37	Group 2	17	*Mortierella verticillata*	"humilis-verticillata complex"	*Mortierella verticillata*	CBS 315.52	JX975943	MF423522	548	0	100.0
YTM181	Group 2	17	*Mortierella verticillata*	"humilis-verticillata complex"	*Mortierella verticillata*	CBS 315.52	JX975943	MF423652	533	0	100.0
YTM222	Group 2	17	*Mortierella verticillata*	"humilis-verticillata complex"	*Mortierella verticillata*	CBS 315.52	JX975943	MF423691	548	0	100.0
YTM226	Group 2	17	*Mortierella verticillata*	"humilis-verticillata complex"	*Mortierella verticillata*	CBS 315.52	JX975943	MF423695	548	0	100.0
YTM22	Group 2	18	*Mortierella horticola*	"minutissima-horticola complex"	*Mortierella horticola*	CBS 869.68	JX976058	MF423511	550	0	99.8
YTM78	Group 2	18	*Mortierella horticola*	"minutissima-horticola complex"	*Mortierella horticola*	CBS 869.68	JX976058	MF423561	519	0	99.8
YTM79	Group 2	18	*Mortierella horticola*	"minutissima-horticola complex"	*Mortierella horticola*	CBS 869.68	JX976058	MF423562	519	0	100.0
YTM80	Group 2	18	*Mortierella horticola*	"minutissima-horticola complex"	*Mortierella horticola*	CBS 869.68	JX976058	MF423563	519	0	100.0
YTM82	Group 2	18	*Mortierella horticola*	"minutissima-horticola complex"	*Mortierella horticola*	CBS 869.68	JX976058	MF423565	519	0	100.0
YTM83	Group 2	18	*Mortierella horticola*	"minutissima-horticola complex"	*Mortierella horticola*	CBS 869.68	JX976058	MF423566	540	0	100.0
YTM84	Group 2	18	*Mortierella horticola*	"minutissima-horticola complex"	*Mortierella horticola*	CBS 869.68	JX976058	MF423567	519	0	100.0
YTM85	Group 2	18	*Mortierella horticola*	"minutissima-horticola complex"	*Mortierella horticola*	CBS 869.68	JX976058	MF423568	519	0	100.0
YTM86	Group 2	18	*Mortierella horticola*	"minutissima-horticola complex"	*Mortierella horticola*	CBS 869.68	JX976058	MF423569	519	0	99.8
YTM218	Group 2	18	*Mortierella horticola*	"minutissima-horticola complex"	*Mortierella horticola*	CBS 869.68	JX976058	MF423688	557	0	99.8
CBS277.71	Group 2	19	*Mortierella minutissima*	"minutissima-horticola complex"	*Mortierella minutissima*	CBS 277.71	JX975938	JX975938	-	-	-
CBS278.71	Group 2	19	*Mortierella minutissima*	"minutissima-horticola complex"	*Mortierella minutissima*	FSU 10804	JX976020	MF403051	520	0	100.0
YTM215	Group 2	19	*Mortierella minutissima*	"minutissima-horticola complex"	*Mortierella minutissima*	CBS 277.71	JX975938	MF423686	547	0	100.0
YTM219	Group 2	19	*Mortierella minutissima*	"minutissima-horticola complex"	*Mortierella minutissima*	CBS 277.71	JX975938	MF423689	547	0	100.0
YTM23	Group 2	20	*Mortierella* sp. "zonata"	"minutissima-horticola complex"	*Mortierella* sp. "zonata"	CBS 863.68	JX975888	MF423512	522	0	100.0
YTM160	Group 2	20	*Mortierella* sp. "zonata"	"minutissima-horticola complex"	*Mortierella* sp. "zonata"	CBS 863.68	JX975888	MF423631	522	0	100.0
YTM7	Group 3	21	*Mortierella calciphila*	*Mortierella calciphila*	*Mortierella calciphila*	CBS 140728	KT964845	MF423497	575	0	93.6
YTM30	Group 3	21	*Mortierella calciphila*	*Mortierella calciphila*	*Mortierella calciphila*	CBS 140728	KT964845	MF423519	554	0	98.6
YTM97	Group 3	21	*Mortierella calciphila*	*Mortierella calciphila*	*Mortierella calciphila*	CBS 140728	KT964845	MF423580	556	0	97.9
YTM21	Group 3	22	*Mortierella gemmifera*	*Mortierella gemmifera*	*Mortierella gemmifera*	CBS 383.85	JX976121	MF423510	569	0	99.6
YTM38	Group 3	22	*Mortierella gemmifera*	*Mortierella gemmifera*	*Mortierella gemmifera*	CBS 383.85	JX976121	MF423523	536	0	96.3
YTM129	Group 3	22	*Mortierella gemmifera*	*Mortierella gemmifera*	*Mortierella gemmifera*	CBS 383.85	JX976121	MF423601	571	0	97.1
YTM132	Group 3	22	*Mortierella gemmifera*	*Mortierella gemmifera*	*Mortierella gemmifera*	CBS 383.85	JX976121	MF423604	567	0	97.1
YTM158	Group 3	22	*Mortierella gemmifera*	*Mortierella gemmifera*	*Mortierella gemmifera*	CBS 383.85	JX976121	MF423629	557	0	97.0
YTM180	Group 3	23	*Mortierella kuhlmanii*	*Mortierella kuhlmanii*	*Mortierella kuhlmanii*	CBS 272.71	JX975964	MF423651	534	0	99.6
YTM182	Group 3	23	*Mortierella kuhlmanii*	*Mortierella kuhlmanii*	*Mortierella kuhlmanii*	CBS 272.71	JX975964	MF423653	549	0	99.5
YTM183	Group 3	23	*Mortierella kuhlmanii*	*Mortierella kuhlmanii*	*Mortierella kuhlmanii*	CBS 272.71	JX975964	MF423654	551	0	99.6
YTM1	Group 4	24	*Dissophora decumbens*	*Dissophora decumbens*	*Mortierella elasson*	CBS 220.29	HQ630368	MF423492	542	0	99.4
YTM60	Group 4	25	*Gamsiella multidivaricata*	*Gamsiella multidivaricata*	*Gamsiella multidivaricata*	CBS 227.78	JX975871	MF423545	588	0	96.1
CBS857.70	Group 4	26	*Mortierella globulifera*	*Mortierella globulifera*	*Mortierella globulifera*	CBS 857.70	JX975910	JX975910	-	-	-
CBS858.70	Group 4	26	*Mortierella globulifera*	*Mortierella globulifera*	*Mortierella globulifera*	CBS 858.70	JX975915	JX975915	-	-	-
YTM42	Group 4	26	*Mortierella globulifera*	*Mortierella globulifera*	*Mortierella globulifera*	CBS 857.70	JX975910	MF423527	532	0	99.4
YTM44	Group 4	26	*Mortierella globulifera*	*Mortierella globulifera*	*Mortierella globulifera*	CBS 857.70	JX975910	MF423529	532	0	99.6
YTM45	Group 4	26	*Mortierella globulifera*	*Mortierella globulifera*	*Mortierella globulifera*	CBS 857.70	JX975910	MF423530	556	0	99.6
YTM93	Group 4	26	*Mortierella globulifera*	*Mortierella globulifera*	*Mortierella globulifera*	CBS 108.68	JX975847	MF423576	563	0	98.2

YTM209	Group 4	26	*Mortierella globulifera*	*Mortierella globulifera*	*Mortierella globulifera*	CBS 857.70	JX975910	MF423680	574	0	99.5
YTM115	Group 5	27	*Mortierella ambigua*	*Mortierella ambigua*	*Mortierella ambigua*	CBS 474.96	JX976056	MF423590	614	0	100.0
YTM69	Group 5	28	*Mortierella capitata*	*Mortierella capitata*	*Mortierella capitata*	CBS 293.96	JX976123	MF423553	549	0	100.0
YTM70	Group 5	28	*Mortierella capitata*	*Mortierella capitata*	*Mortierella capitata*	CBS 293.96	JX976123	MF423554	549	0	100.0
YTM71	Group 5	28	*Mortierella capitata*	*Mortierella capitata*	*Mortierella capitata*	CBS 293.96	JX976123	MF423555	546	0	100.0
YTM72	Group 5	28	*Mortierella capitata*	*Mortierella capitata*	*Mortierella capitata*	CBS 293.96	JX976123	MF423556	549	0	100.0
YTM140	Group 5	28	*Mortierella capitata*	*Mortierella capitata*	*Mortierella capitata*	CBS 293.96	JX976123	MF423611	574	0	100.0
YTM141	Group 5	28	*Mortierella capitata*	*Mortierella capitata*	*Mortierella capitata*	CBS 293.96	JX976123	MF423612	573	0	100.0
YTM142	Group 5	28	*Mortierella capitata*	*Mortierella capitata*	*Mortierella capitata*	CBS 293.96	JX976123	MF423613	574	0	100.0
YTM143	Group 5	28	*Mortierella capitata*	*Mortierella capitata*	*Mortierella capitata*	CBS 293.96	JX976123	MF423614	576	0	100.0
YTM144	Group 5	28	*Mortierella capitata*	*Mortierella capitata*	*Mortierella capitata*	CBS 293.96	JX976123	MF423615	576	0	100.0
YTM145	Group 5	28	*Mortierella capitata*	*Mortierella capitata*	*Mortierella capitata*	CBS 293.96	JX976123	MF423616	576	0	100.0
YTM135	Group 5	29	*Mortierella oedorhiza* **	*Mortierella oedorhiza* **	*Mortierella rostafinskii*	CBS 522.70	JX975885	MF423607	581	2.04E-146	85.3
NBRC112302	Group 5	30	*Mortierella wolfii*	*Mortierella wolfii*	*Mortierella wolfii*	CBS 209.69	HQ630303	MH802521	580	0	100.0
NBRC112303	Group 5	30	*Mortierella wolfii*	*Mortierella wolfii*	*Mortierella wolfii*	CBS 651.93	JX975904	MH802520	579	0	100.0
YTM104	Group 5	31	*Mortierella* sp. 4	*Mortierella* sp. 4	*Mortierella ambigua*	CBS 521.80	JX976120	MF423584	600	0	98.0
YTM108	Group 5	31	*Mortierella* sp. 4	*Mortierella* sp. 4	*Mortierella ambigua*	CBS 521.80	JX976120	MF423585	600	0	98.0
YTM168	Group 5	31	*Mortierella* sp. 4	*Mortierella* sp. 4	*Mortierella ambigua*	CBS 457.66	JX976041	MF423639	569	0	88.9
YTM169	Group 5	31	*Mortierella* sp. 4	*Mortierella* sp. 4	*Mortierella ambigua*	CBS 521.80	JX976120	MF423640	570	0	89.1
YTM49	Group 5	32	*Mortierella* sp. 5	*Mortierella* sp. 5	*Mortierella ambigua*	CBS 521.80	JX976120	MF423534	575	0	92.8
YTM50	Group 5	32	*Mortierella* sp. 5	*Mortierella* sp. 5	*Mortierella ambigua*	CBS 521.80	JX976120	MF423535	585	0	93.0
YTM73	Group 5	32	*Mortierella* sp. 5	*Mortierella* sp. 5	*Mortierella ambigua*	CBS 521.80	JX976120	MF423557	559	0	93.0
YTM53	Group 5	33	*Mortierella* sp. 6	*Mortierella* sp. 6	*Mortierella ambigua*	CBS 521.80	JX976120	MF423538	583	1.27E-123	86.4
YTM6	Group 5	34	*Mortierella* sp. 7	*Mortierella* sp. 7	*Mortierella capitata*	CBS 293.96	JX976123	MF423496	583	7.26E-156	88.6
YTM74	Group 5	35	*Mortierella* sp. 8	*Mortierella* sp. 8	*Mortierella capitata*	CBS 293.96	JX976123	MF423558	577	0	92.8
YTM75	Group 5	35	*Mortierella* sp. 8	*Mortierella* sp. 8	*Mortierella capitata*	CBS 293.96	JX976123	MF423559	577	0	92.8
YTM76	Group 5	35	*Mortierella* sp. 8	*Mortierella* sp. 8	*Mortierella capitata*	CBS 293.96	JX976123	MF423560	556	0	92.8
YTM96	Group 5	35	*Mortierella* sp. 8	*Mortierella* sp. 8	*Mortierella capitata*	CBS 293.96	JX976123	MF423579	610	0	93.1
YTM167	Group 5	36	*Mortierella* sp. 9	*Mortierella* sp. 9	*Mortierella capitata*	CBS 293.96	JX976123	MF423638	573	1.53E-157	89.7
YTM3	Group 6	37	*Mortierella alpina*	"alpina complex"	*Mortierella alpina*	CBS 110518	JX975906	MF423493	585	0	99.1
YTM4	Group 6	37	*Mortierella alpina*	"alpina complex"	*Mortierella alpina*	CBS 110518	JX975906	MF423494	586	0	100.0
YTM5	Group 6	37	*Mortierella alpina*	"alpina complex"	*Mortierella alpina*	CBS 110518	JX975906	MF423495	585	0	99.1
YTM25	Group 6	37	*Mortierella alpina*	"alpina complex"	*Mortierella alpina*	FSU 10715	JX976080	MF423514	615	0	100.0
YTM27	Group 6	37	*Mortierella alpina*	"alpina complex"	*Mortierella alpina*	FSU 10696	JX976108	MF423516	577	0	99.5
YTM40	Group 6	37	*Mortierella alpina*	"alpina complex"	*Mortierella alpina*	FSU 10696	JX976108	MF423525	583	0	99.5
YTM81	Group 6	37	*Mortierella alpina*	"alpina complex"	*Mortierella alpina*	FSU 10715	JX976080	MF423564	547	0	100.0
YTM87	Group 6	37	*Mortierella alpina*	"alpina complex"	*Mortierella alpina*	FSU 10715	JX976080	MF423570	571	0	100.0
YTM88	Group 6	37	*Mortierella alpina*	"alpina complex"	*Mortierella alpina*	FSU 10715	JX976080	MF423571	547	0	100.0
YTM89	Group 6	37	*Mortierella alpina*	"alpina complex"	*Mortierella alpina*	FSU 10715	JX976080	MF423572	547	0	100.0
YTM117	Group 6	37	*Mortierella alpina*	"alpina complex"	*Mortierella alpina*	CBS 529.72	JX976124	MF423591	590	0	96.1
YTM120	Group 6	37	*Mortierella alpina*	"alpina complex"	*Mortierella alpina*	FSU 8722	JX975961	MF423593	590	0	96.0
YTM156	Group 6	37	*Mortierella alpina*	"alpina complex"	*Mortierella alpina*	CBS 529.72	JX976124	MF423627	586	0	96.1
YTM159	Group 6	37	*Mortierella alpina*	"alpina complex"	*Mortierella alpina*	FSU 10522	JX975930	MF423630	477	7.90E-130	87.3
YTM173	Group 6	37	*Mortierella alpina*	"alpina complex"	*Mortierella alpina*	CBS 110518	JX975906	MF423644	548	0	98.9
YTM188	Group 6	37	*Mortierella alpina*	"alpina complex"	*Mortierella alpina*	FSU 10715	JX976080	MF423659	547	0	100.0
YTM189	Group 6	37	*Mortierella alpina*	"alpina complex"	*Mortierella alpina*	FSU 10715	JX976080	MF423660	549	0	100.0
YTM202	Group 6	37	*Mortierella alpina*	"alpina complex"	*Mortierella alpina*	CBS 387.71	JX976038	MF423673	549	0	100.0
YTM203	Group 6	37	*Mortierella alpina*	"alpina complex"	*Mortierella alpina*	CBS 387.71	JX976038	MF423674	588	0	100.0
YTM204	Group 6	37	*Mortierella alpina*	"alpina complex"	*Mortierella alpina*	CBS 110518	JX975906	MF423675	548	0	98.7
YTM205	Group 6	37	*Mortierella alpina*	"alpina complex"	*Mortierella alpina*	FSU 10696	JX976108	MF423676	570	0	99.3
NBRC100829	Group 6	38	*Mortierella hypsicladia*	*Mortierella hypsicladia*	*Mortierella hypsicladia*	CBS 116203	JX975872	MH802523	586	0	100.0

YTM62	Group 6	39	*Mortierella oligospora*	*Mortierella oligospora*	*Mortierella oligospora*	CBS 101758	JX976032	MF423547	575	0	100.0
YTM24	Group 6	40	*Mortierella polycephala*	*Mortierella polycephala*	*Mortierella polycephala*	CBS 328.72	JX976102	MF423513	574	0	99.8
YTM63	Group 6	40	*Mortierella polycephala*	*Mortierella polycephala*	*Mortierella polycephala*	CBS 328.72	JX976102	MF423548	545	0	100.0
YTM146	Group 6	41	*Mortierella* sp. 10	*Mortierella* sp. 10	*Mortierella bisporalis*	FSU 9675	JX975953	MF423617	604	0	96.0
YTM147	Group 6	41	*Mortierella* sp. 10	*Mortierella* sp. 10	*Mortierella bisporalis*	FSU 9675	JX975953	MF423618	601	0	96.0
YTM148	Group 6	41	*Mortierella* sp. 10	*Mortierella* sp. 10	*Mortierella bisporalis*	FSU 9675	JX975953	MF423619	608	0	96.1
YTM149	Group 6	41	*Mortierella* sp. 10	*Mortierella* sp. 10	*Mortierella bisporalis*	FSU 9675	JX975953	MF423620	609	0	96.1
YTM153	Group 6	41	*Mortierella* sp. 10	*Mortierella* sp. 10	*Mortierella bisporalis*	FSU 9675	JX975953	MF423624	604	0	96.0
YTM154	Group 6	41	*Mortierella* sp. 10	*Mortierella* sp. 10	*Mortierella bisporalis*	FSU 9675	JX975953	MF423625	608	0	96.1
YTM155	Group 6	41	*Mortierella* sp. 10	*Mortierella* sp. 10	*Mortierella bisporalis*	FSU 9675	JX975953	MF423626	604	0	96.0
YTM150	Group 6	42	*Mortierella* sp. 11	*Mortierella* sp. 11	*Mortierella bisporalis*	FSU 9675	JX975953	MF423621	600	0	97.3
YTM151	Group 6	42	*Mortierella* sp. 11	*Mortierella* sp. 11	*Mortierella bisporalis*	FSU 9675	JX975953	MF423622	594	0	97.3
YTM152	Group 6	42	*Mortierella* sp. 11	*Mortierella* sp. 11	*Mortierella bisporalis*	FSU 9675	JX975953	MF423623	588	0	97.3
YTM100	Group 7	43	*Mortierella biramosa*	*Mortierella biramosa*	*Mortierella biramosa*	CBS 370.95	JX976094	MF423583	538	0	99.4
YTM111	Group 7	43	*Mortierella biramosa*	*Mortierella biramosa*	*Mortierella biramosa*	CBS 370.95	JX976094	MF423587	538	0	99.4
CBS121.71	Group 7	44	*Mortierella elongata*	"elongata complex"	*Mortierella elongata*	CBS 126.71	JX976101	MF403050	526	0	100.0
CBS122.71	Group 7	44	*Mortierella elongata*	"elongata complex"	*Mortierella elongata*	CBS 122.71	JX976000	JX976000	-	-	-
FMR13-2	Group 7	44	*Mortierella elongata*	"elongata complex"	*Mortierella elongata*	CBS 126.71	JX976101	AB542093	554	0	100.0
FMR23-1	Group 7	44	*Mortierella elongata*	"elongata complex"	*Mortierella elongata*	CBS 126.71	JX976101	AB542099	554	0	99.8
FMR23-6	Group 7	44	*Mortierella elongata*	"elongata complex"	*Mortierella elongata*	CBS 126.71	JX976101	AB542104	554	0	100.0
FMR23-9	Group 7	44	*Mortierella elongata*	"elongata complex"	*Mortierella elongata*	CBS 126.71	JX976101	AB542107	554	0	100.0
YTM17	Group 7	44	*Mortierella elongata*	"elongata complex"	*Mortierella elongata*	CBS 126.71	JX976101	MF423506	560	0	100.0
YTM18	Group 7	44	*Mortierella elongata*	"elongata complex"	*Mortierella elongata*	CBS 276.89	JX976111	MF423507	546	0	99.6
YTM19	Group 7	44	*Mortierella elongata*	"elongata complex"	*Mortierella elongata*	CBS 276.89	JX976111	MF423508	561	0	99.8
YTM43	Group 7	44	*Mortierella elongata*	"elongata complex"	*Mortierella elongata*	CBS 126.71	JX976101	MF423528	548	0	100.0
YTM54	Group 7	44	*Mortierella elongata*	"elongata complex"	*Mortierella elongata*	CBS 276.89	JX976111	MF423539	548	0	99.8
YTM55	Group 7	44	*Mortierella elongata*	"elongata complex"	*Mortierella elongata*	CBS 276.89	JX976111	MF423540	548	0	99.8
YTM98	Group 7	44	*Mortierella elongata*	"elongata complex"	*Mortierella elongata*	CBS 126.71	JX976101	MF423581	559	0	99.8
YTM99	Group 7	44	*Mortierella elongata*	"elongata complex"	*Mortierella elongata*	CBS 126.71	JX976101	MF423582	561	0	99.8
YTM122	Group 7	44	*Mortierella elongata*	"elongata complex"	*Mortierella elongata*	CBS 126.71	JX976101	MF423594	559	0	100.0
YTM138	Group 7	44	*Mortierella elongata*	"elongata complex"	*Mortierella elongata*	CBS 126.71	JX976101	MF423609	554	0	100.0
YTM139	Group 7	44	*Mortierella elongata*	"elongata complex"	*Mortierella elongata*	CBS 126.71	JX976101	MF423610	548	0	100.0
YTM162	Group 7	44	*Mortierella elongata*	"elongata complex"	*Mortierella elongata*	CBS 126.71	JX976101	MF423633	526	0	100.0
YTM163	Group 7	44	*Mortierella elongata*	"elongata complex"	*Mortierella elongata*	CBS 126.71	JX976101	MF423634	526	0	100.0
YTM164	Group 7	44	*Mortierella elongata*	"elongata complex"	*Mortierella elongata*	CBS 126.71	JX976101	MF423635	526	0	100.0
YTM165	Group 7	44	*Mortierella elongata*	"elongata complex"	*Mortierella elongata*	CBS 126.71	JX976101	MF423636	526	0	100.0
YTM170	Group 7	44	*Mortierella elongata*	"elongata complex"	*Mortierella elongata*	CBS 126.71	JX976101	MF423641	526	0	100.0
YTM174	Group 7	44	*Mortierella elongata*	"elongata complex"	*Mortierella elongata*	CBS 126.71	JX976101	MF423645	526	0	100.0
YTM190	Group 7	44	*Mortierella elongata*	"elongata complex"	*Mortierella elongata*	CBS 126.71	JX976101	MF423661	526	0	100.0
YTM191	Group 7	44	*Mortierella elongata*	"elongata complex"	*Mortierella elongata*	CBS 126.71	JX976101	MF423662	539	0	100.0
YTM192	Group 7	44	*Mortierella elongata*	"elongata complex"	*Mortierella elongata*	CBS 126.71	JX976101	MF423663	538	0	100.0
YTM193	Group 7	44	*Mortierella elongata*	"elongata complex"	*Mortierella elongata*	CBS 126.71	JX976101	MF423664	526	0	100.0
YTM194	Group 7	44	*Mortierella elongata*	"elongata complex"	*Mortierella elongata*	CBS 126.71	JX976101	MF423665	526	0	100.0
YTM195	Group 7	44	*Mortierella elongata*	"elongata complex"	*Mortierella elongata*	CBS 126.71	JX976101	MF423666	526	0	100.0
YTM199	Group 7	44	*Mortierella elongata*	"elongata complex"	*Mortierella elongata*	CBS 126.71	JX976101	MF423670	526	0	100.0
YTM201	Group 7	44	*Mortierella elongata*	"elongata complex"	*Mortierella elongata*	CBS 126.71	JX976101	MF423672	537	0	100.0
YTM206	Group 7	44	*Mortierella elongata*	"elongata complex"	*Mortierella elongata*	CBS 126.71	JX976101	MF423677	546	0	100.0
YTM207	Group 7	44	*Mortierella elongata*	"elongata complex"	*Mortierella elongata*	CBS 126.71	JX976101	MF423678	527	0	100.0
YTM210	Group 7	44	*Mortierella elongata*	"elongata complex"	*Mortierella elongata*	CBS 126.71	JX976101	MF423681	548	0	100.0
YTM178	Group 7	45	*Mortierella exigua*	*Mortierella exigua*	*Mortierella exigua*	CBS 655.68	JX976047	MF423649	517	0	99.8
YTM172	Group 7	46	*Mortierella fatshederae*	*Mortierella fatshederae*	*Mortierella fatshederae*	CBS 388.71	JX976003	MF423643	549	0	98.0

YTM123	Group 7	47	*Mortierella gamsii*	*Mortierella gamsii*	*Mortierella gamsii*	CBS 110630	JX976106	MF423595	557	0	100.0
YTM124	Group 7	47	*Mortierella gamsii*	*Mortierella gamsii*	*Mortierella gamsii*	FSU 2057	JX976118	MF423596	554	0	99.6
YTM127	Group 7	47	*Mortierella gamsii*	*Mortierella gamsii*	*Mortierella gamsii*	CBS 110630	JX976106	MF423599	557	0	100.0
YTM131	Group 7	47	*Mortierella gamsii*	*Mortierella gamsii*	*Mortierella gamsii*	FSU 2057	JX976118	MF423603	527	0	99.8
NBRC109920	Group 7	48	*Mortierella thereuopodae*	*Mortierella thereuopodae*	*Mortierella thereuopodae*	NBRC 109920	AB862878	AB862878	-	-	-
NBRC109921	Group 7	48	*Mortierella thereuopodae*	*Mortierella thereuopodae*	*Mortierella thereuopodae*	NBRC 109921	AB862879	AB862879	-	-	-
YTM68	Group 7	49	*Mortierella zonata*	"zonata complex"	*Mortierella zonata*	FSU 2735	JX976103	MF423552	538	0	96.9
YTM10	Group 7	50	*Mortierella zychae*	*Mortierella zychae*	*Mortierella zychae*	FSU 719	JX976128	MF423500	521	0	100.0
YTM12	Group 7	50	*Mortierella zychae*	*Mortierella zychae*	*Mortierella zychae*	FSU 719	JX976128	MF423502	538	0	98.5
YTM29	Group 7	50	*Mortierella zychae*	*Mortierella zychae*	*Mortierella zychae*	FSU 719	JX976128	MF423518	538	0	98.5
YTM65	Group 7	50	*Mortierella zychae*	*Mortierella zychae*	*Mortierella zychae*	FSU 719	JX976128	MF423550	520	0	98.8
YTM20	Group 7	51	*Mortierella* sp. CBS 118520	"elongata complex"	*Mortierella* sp. CBS 118520	CBS 118520	JX975936	MF423509	543	0	98.7
YTM134	Group 7	52	*Mortierella* sp. 12	*Mortierella* sp. 12	*Mortierella fluviae*	EML-YR25716-2	KX227756	MF423606	554	0	98.0
YTM137	Group 7	52	*Mortierella* sp. 12	*Mortierella* sp. 12	*Mortierella fluviae*	EML-YR25716-2	KX227756	MF423608	550	0	98.0
NBRC8569	Group 7	52	*Mortierella* sp. 12	*Mortierella* sp. 12	*Mortierella gamsii*	CBS 552.73	JX975984	MH802518	523	0	97.9
YTM196	Group 7	53	*Mortierella* sp. 13	*Mortierella* sp. 13	*Mortierella schmuckeri*	CBS 156.78	JX975854	MF423667	518	0	98.7
YTM197	Group 7	53	*Mortierella* sp. 13	*Mortierella* sp. 13	*Mortierella schmuckeri*	CBS 156.78	JX975854	MF423668	533	0	98.7
YTM198	Group 7	53	*Mortierella* sp. 13	*Mortierella* sp. 13	*Mortierella schmuckeri*	CBS 156.78	JX975854	MF423669	518	0	98.7
YTM200	Group 7	53	*Mortierella* sp. 13	*Mortierella* sp. 13	*Mortierella schmuckeri*	CBS 156.78	JX975854	MF423671	544	0	98.7
YTM161	Group 7	54	*Mortierella* sp. 14	*Mortierella* sp. 14	*Mortierella claussenii*	CBS 790.85	JX976012	MF423632	520	0	98.8
YTM8	Group 7	55	*Mortierella* sp. 15	*Mortierella* sp. 15	*Mortierella exigua*	CBS 358.76	JX976113	MF423498	552	0	100.0
YTM9	Group 7	55	*Mortierella* sp. 15	*Mortierella* sp. 15	*Mortierella exigua*	CBS 358.76	JX976113	MF423499	552	0	100.0
YTM26	Group 7	55	*Mortierella* sp. 15	*Mortierella* sp. 15	*Mortierella exigua*	CBS 358.76	JX976113	MF423515	546	0	100.0
YTM171	Group 7	55	*Mortierella* sp. 15	*Mortierella* sp. 15	*Mortierella exigua*	CBS 358.76	JX976113	MF423642	518	0	100.0
YTM176	Group 7	55	*Mortierella* sp. 15	*Mortierella* sp. 15	*Mortierella exigua*	CBS 358.76	JX976113	MF423647	540	0	100.0
YTM177	Group 7	55	*Mortierella* sp. 15	*Mortierella* sp. 15	*Mortierella exigua*	CBS 358.76	JX976113	MF423648	518	0	100.0
YTM110	Group 7	56	*Mortierella* sp. 16	*Mortierella* sp. 16	*Mortierella fluviae*	EML-YR25716-2	KX227756	MF423586	550	0	99.5
YTM112	Group 7	56	*Mortierella* sp. 16	*Mortierella* sp. 16	*Mortierella gamsii*	CBS 552.73	JX975984	MF423588	555	0	99.5
YTM113	Group 7	56	*Mortierella* sp. 16	*Mortierella* sp. 16	*Mortierella gamsii*	CBS 552.73	JX975984	MF423589	555	0	99.5
YTM179	Group 7	56	*Mortierella* sp. 16	*Mortierella* sp. 16	*Mortierella gamsii*	CBS 551.73	JX976079	MF423650	524	0	99.4
YTM184	Group 7	56	*Mortierella* sp. 16	*Mortierella* sp. 16	*Mortierella gamsii*	CBS 551.73	JX976079	MF423655	521	0	99.8
YTM185	Group 7	56	*Mortierella* sp. 16	*Mortierella* sp. 16	*Mortierella gamsii*	CBS 551.73	JX976079	MF423656	521	0	99.8
YTM186	Group 7	56	*Mortierella* sp. 16	*Mortierella* sp. 16	*Mortierella gamsii*	CBS 551.73	JX976079	MF423657	521	0	99.6
YTM212	Group 7	57	*Mortierella* sp. 17	*Mortierella* sp. 17	*Mortierella fluviae*	EML-YR25716-2	KX227756	MF423683	574	0	99.8
YTM214	Group 7	57	*Mortierella* sp. 17	*Mortierella* sp. 17	*Mortierella fluviae*	EML-YR25716-2	KX227756	MF423685	541	0	99.8
YTM217	Group 7	57	*Mortierella* sp. 17	*Mortierella* sp. 17	*Mortierella fluviae*	EML-YR25716-2	KX227756	MF423687	541	0	99.8
YTM64	Group 7	58	*Mortierella* sp. 18	*Mortierella* sp. 18	*Mortierella gamsii*	FSU 2057	JX976118	MF423549	598	2.62E-170	85.3
YTM28	Group 7	59	*Mortierella* sp. 19	*Mortierella* sp. 19	*Mortierella gamsii*	FSU 2057	JX976118	MF423517	568	1.94E-166	86.3
YTM41	Group 7	59	*Mortierella* sp. 19	*Mortierella* sp. 19	*Mortierella gamsii*	FSU 2057	JX976118	MF423526	548	5.12E-177	87.5

†Species names were mostly identified by phylogenetic analyses; however, species names for the complex lineages were identified on the basis of blastn searches.

**Mortierella sugadairana* is a new species described by Takashima *et al*. (2018) examined by isolates NBRC 104553, YTM39, and YTM128.

***Mortierella oedorhiza* is a species re-described by Takashima *et al*. (submitted to Mycoscience) examined by the isolate YTM135.

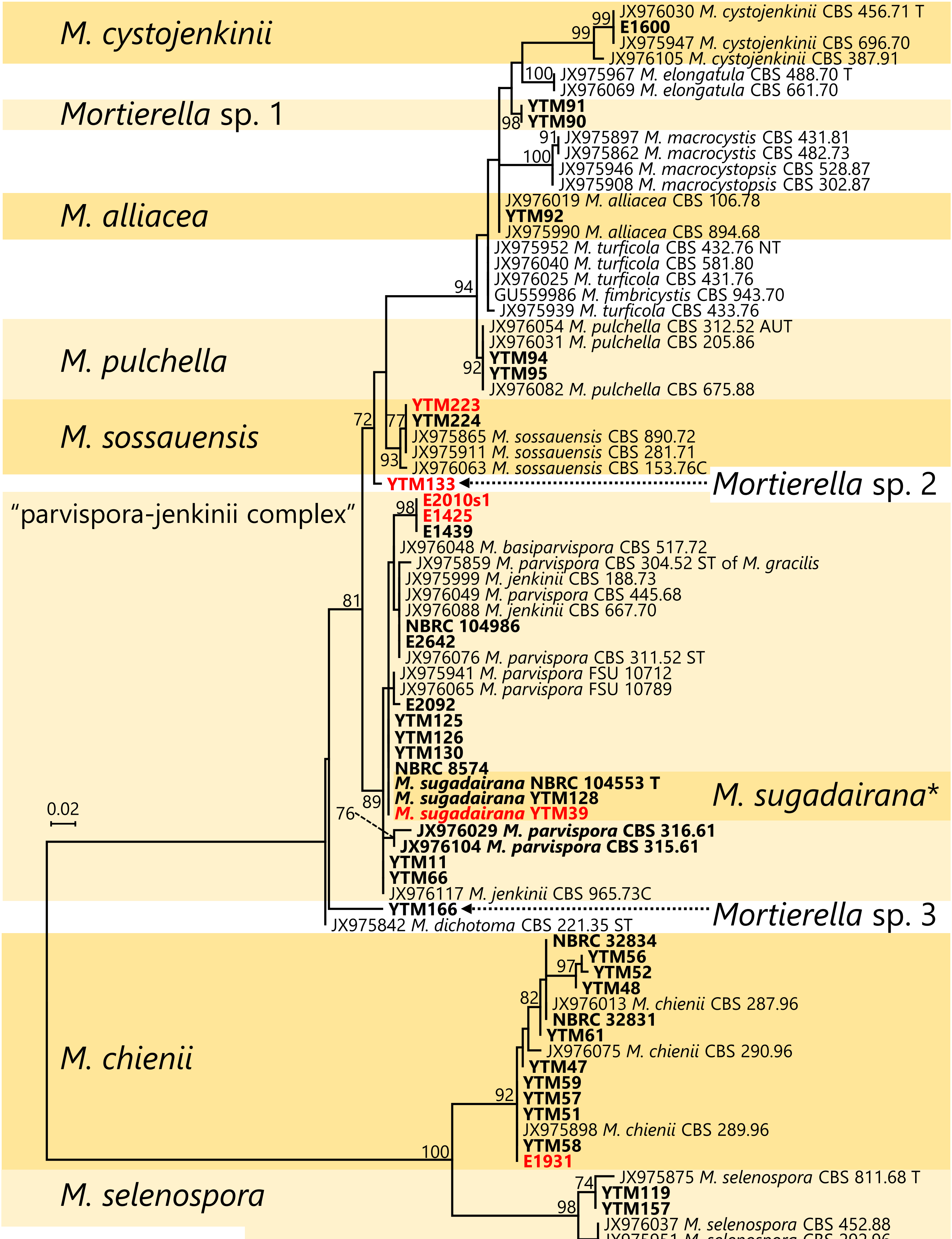

M. cystojenkinii
Mortierella sp. 1
M. alliacea
M. pulchella
M. sossauensis
"parvispora-jenkinii complex"
M. chienii
M. selenospora
Mortierella sp. 2
Mortierella sp. 3
M. sugadairana*
0.02
JX976030 M. cystojenkinii CBS 456.71 T
E1600
JX975947 M. cystojenkinii CBS 696.70
JX976105 M. cystojenkinii CBS 387.91
JX975967 M. elongatula CBS 488.70 T
JX976069 M. elongatula CBS 661.70
YTM91
YTM90
JX975897 M. macrocystis CBS 431.81
JX975862 M. macrocystis CBS 482.73
JX975946 M. macrocystopsis CBS 528.87
JX975908 M. macrocystopsis CBS 302.87
JX976019 M. alliacea CBS 106.78
YTM92
JX975990 M. alliacea CBS 894.68
JX975952 M. turficola CBS 432.76 NT
JX976040 M. turficola CBS 581.80
JX976025 M. turficola CBS 431.76
GU559986 M. fimbricystis CBS 943.70
JX975939 M. turficola CBS 433.76
JX976054 M. pulchella CBS 312.52 AUT
JX976031 M. pulchella CBS 205.86
YTM94
YTM95
JX976082 M. pulchella CBS 675.88
YTM223
YTM224
JX975865 M. sossauensis CBS 890.72
JX975911 M. sossauensis CBS 281.71
JX976063 M. sossauensis CBS 153.76C
YTM133
E2010s1
E1425
E1439
JX976048 M. basiparvispora CBS 517.72
JX975859 M. parvispora CBS 304.52 ST of M. gracilis
JX975999 M. jenkinii CBS 188.73
JX976049 M. parvispora CBS 445.68
JX976088 M. jenkinii CBS 667.70
NBRC 104986
E2642
JX976076 M. parvispora CBS 311.52 ST
JX975941 M. parvispora FSU 10712
JX976065 M. parvispora FSU 10789
E2092
YTM125
YTM126
YTM130
NBRC 8574
M. sugadairana NBRC 104553 T
M. sugadairana YTM128
M. sugadairana YTM39
JX976029 M. parvispora CBS 316.61
JX976104 M. parvispora CBS 315.61
YTM11
YTM66
JX976117 M. jenkinii CBS 965.73C
YTM166
JX975842 M. dichotoma CBS 221.35 ST
NBRC 32834
YTM56
YTM52
YTM48
JX976013 M. chienii CBS 287.96
NBRC 32831
YTM61
JX976075 M. chienii CBS 290.96
YTM47
YTM59
YTM57
YTM51
JX975898 M. chienii CBS 289.96
YTM58
E1931
JX975875 M. selenospora CBS 811.68 T
YTM119
YTM157
JX976037 M. selenospora CBS 452.88
JX975951 M. selenospora CBS 292.96
JX975912 M. selenospora CBS 554.73

Figure S1A

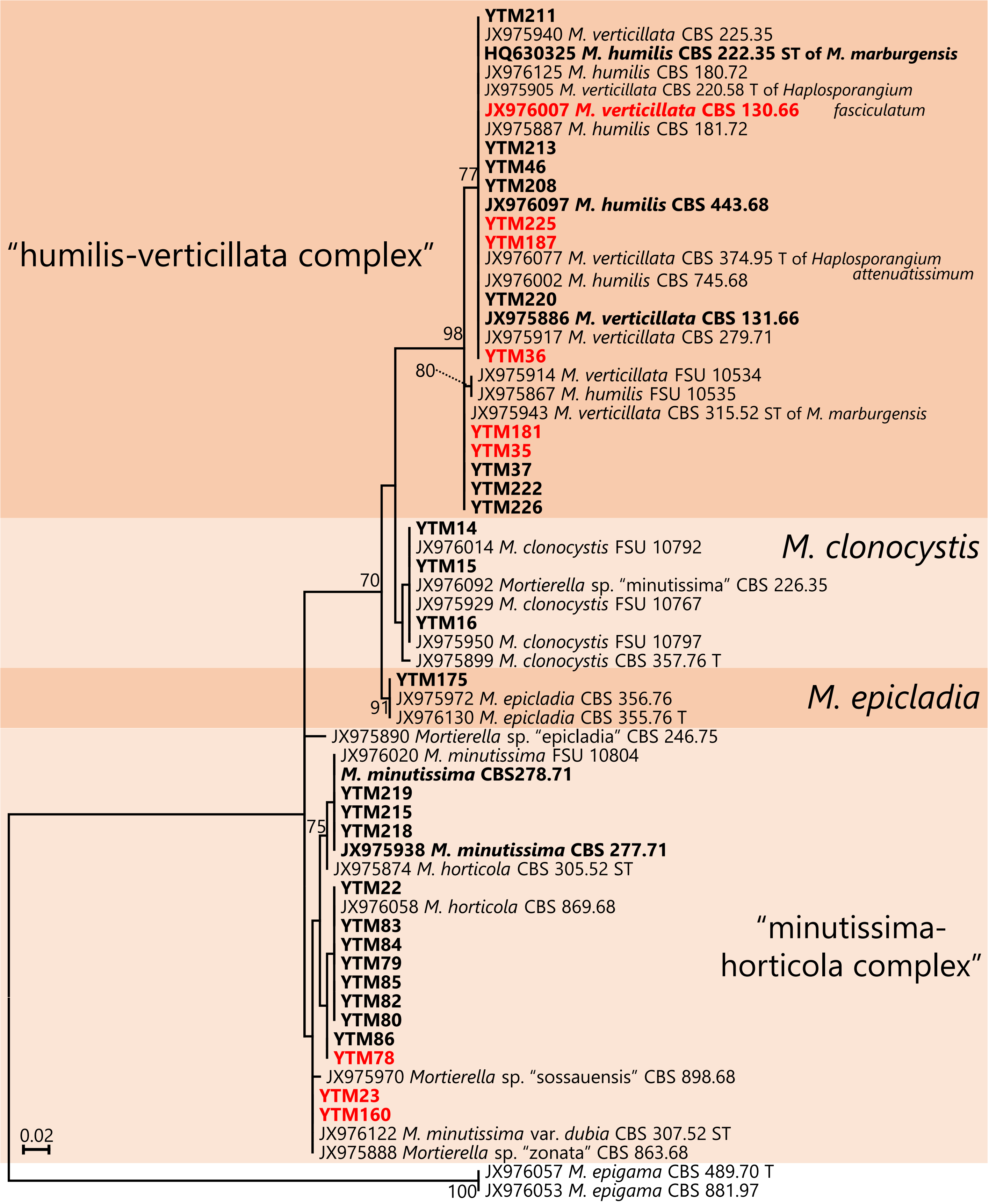

YTM211
JX975940 M. verticillata CBS 225.35
HQ630325 M. humilis CBS 222.35 ST of M. marburgensis
JX976125 M. humilis CBS 180.72
JX975905 M. verticillata CBS 220.58 T of Haplosporangium fasciculatum
JX976007 M. verticillata CBS 130.66
JX975887 M. humilis CBS 181.72
YTM213
YTM46
YTM208
JX976097 M. humilis CBS 443.68
YTM225
YTM187
JX976077 M. verticillata CBS 374.95 T of Haplosporangium attenuatissimum
JX976002 M. humilis CBS 745.68
YTM220
JX975886 M. verticillata CBS 131.66
JX975917 M. verticillata CBS 279.71
YTM36
JX975914 M. verticillata FSU 10534
JX975867 M. humilis FSU 10535
JX975943 M. verticillata CBS 315.52 ST of M. marburgensis
YTM181
YTM35
YTM37
YTM222
YTM226
"humilis-verticillata complex"
77
98
80
70
YTM14
JX976014 M. clonocystis FSU 10792
YTM15
JX976092 Mortierella sp. "minutissima" CBS 226.35
JX975929 M. clonocystis FSU 10767
YTM16
JX975950 M. clonocystis FSU 10797
JX975899 M. clonocystis CBS 357.76 T
M. clonocystis
YTM175
JX975972 M. epicladia CBS 356.76
JX976130 M. epicladia CBS 355.76 T
91
M. epicladia
JX975890 Mortierella sp. "epicladia" CBS 246.75
JX976020 M. minutissima FSU 10804
M. minutissima CBS278.71
YTM219
YTM215
YTM218
JX975938 M. minutissima CBS 277.71
JX975874 M. horticola CBS 305.52 ST
75
YTM22
JX976058 M. horticola CBS 869.68
YTM83
YTM84
YTM79
YTM85
YTM82
YTM80
YTM86
YTM78
JX975970 Mortierella sp. "sossauensis" CBS 898.68
YTM23
YTM160
JX976122 M. minutissima var. dubia CBS 307.52 ST
JX975888 Mortierella sp. "zonata" CBS 863.68
"minutissima-horticola complex"
0.02
JX976057 M. epigama CBS 489.70 T
JX976053 M. epigama CBS 881.97
100

Figure S1B

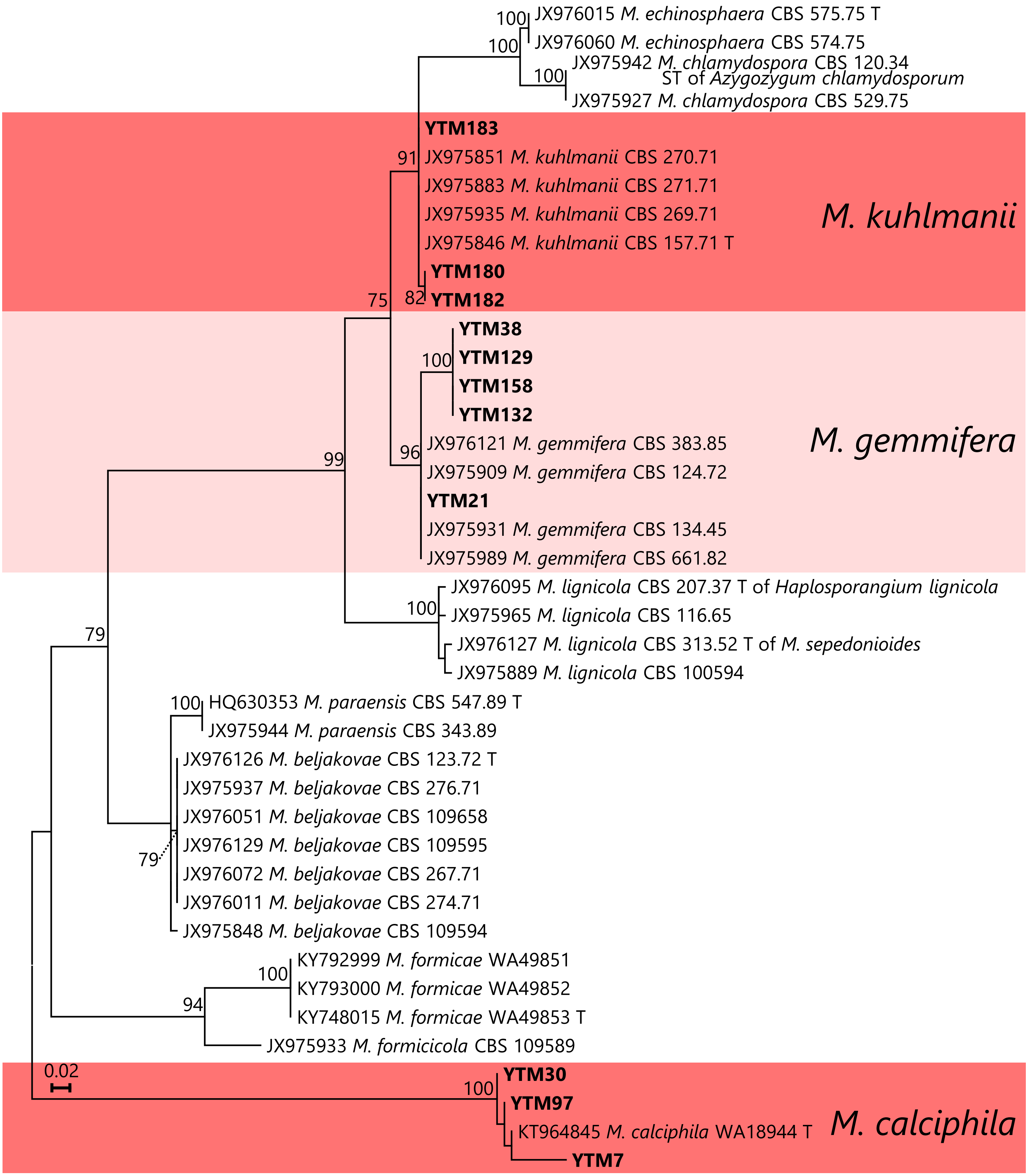
100
JX976015 M. echinosphaera CBS 575.75 T
100
JX976060 M. echinosphaera CBS 574.75
JX975942 M. chlamydospora CBS 120.34
100
ST of Azygozygum chlamydosporum
JX975927 M. chlamydospora CBS 529.75
YTM183
91
JX975851 M. kuhlmanii CBS 270.71
JX975883 M. kuhlmanii CBS 271.71
JX975935 M. kuhlmanii CBS 269.71
JX975846 M. kuhlmanii CBS 157.71 T
M. kuhlmanii
YTM180
75
82
YTM182
YTM38
100
YTM129
YTM158
YTM132
JX976121 M. gemmifera CBS 383.85
96
99
JX975909 M. gemmifera CBS 124.72
M. gemmifera
YTM21
JX975931 M. gemmifera CBS 134.45
JX975989 M. gemmifera CBS 661.82
JX976095 M. lignicola CBS 207.37 T of Haplosporangium lignicola
100
JX975965 M. lignicola CBS 116.65
79
JX976127 M. lignicola CBS 313.52 T of M. sepedonioides
JX975889 M. lignicola CBS 100594
100
HQ630353 M. paraensis CBS 547.89 T
JX975944 M. paraensis CBS 343.89
JX976126 M. beljakovae CBS 123.72 T
JX975937 M. beljakovae CBS 276.71
JX976051 M. beljakovae CBS 109658
JX976129 M. beljakovae CBS 109595
79
JX976072 M. beljakovae CBS 267.71
JX976011 M. beljakovae CBS 274.71
JX975848 M. beljakovae CBS 109594
KY792999 M. formicae WA49851
100
KY793000 M. formicae WA49852
94
KY748015 M. formicae WA49853 T
JX975933 M. formicicola CBS 109589
0.02
YTM30
100
YTM97
KT964845 M. calciphila WA18944 T
M. calciphila
YTM7

Figure S1C

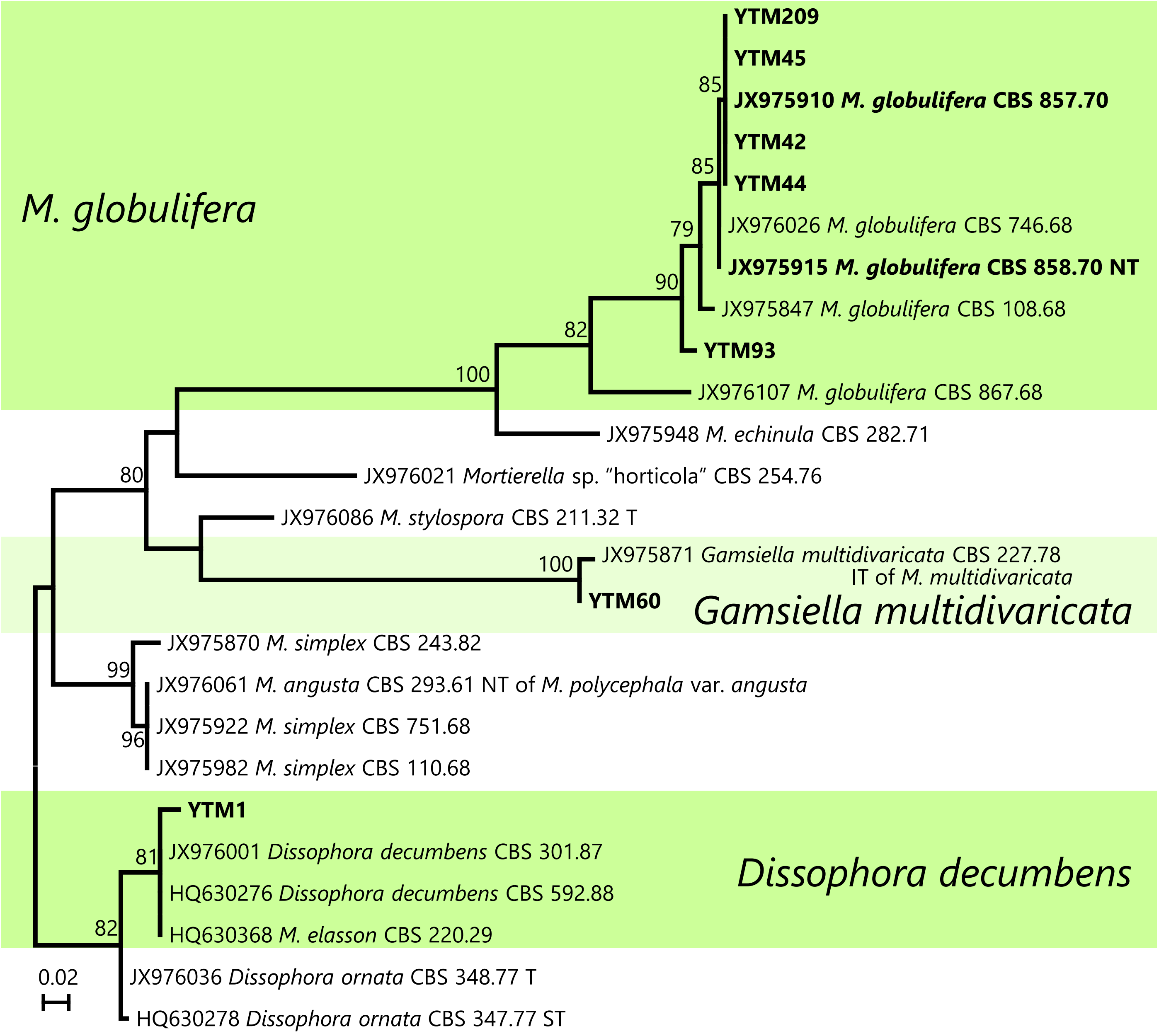
YTM209
YTM45
85
JX975910 M. globulifera CBS 857.70
YTM42
85
YTM44
M. globulifera
JX976026 M. globulifera CBS 746.68
79
JX975915 M. globulifera CBS 858.70 NT
90
JX975847 M. globulifera CBS 108.68
82
YTM93
100
JX976107 M. globulifera CBS 867.68
JX975948 M. echinula CBS 282.71
JX976021 Mortierella sp. "horticola" CBS 254.76
80
JX976086 M. stylospora CBS 211.32 T
100
JX975871 Gamsiella multidivaricata CBS 227.78
IT of M. multidivaricata
YTM60
Gamsiella multidivaricata
JX975870 M. simplex CBS 243.82
99
JX976061 M. angusta CBS 293.61 NT of M. polycephala var. angusta
JX975922 M. simplex CBS 751.68
96
JX975982 M. simplex CBS 110.68
YTM1
81
JX976001 Dissophora decumbens CBS 301.87
HQ630276 Dissophora decumbens CBS 592.88
Dissophora decumbens
82
HQ630368 M. elasson CBS 220.29
0.02
JX976036 Dissophora ornata CBS 348.77 T
HQ630278 Dissophora ornata CBS 347.77 ST

Figure S1D

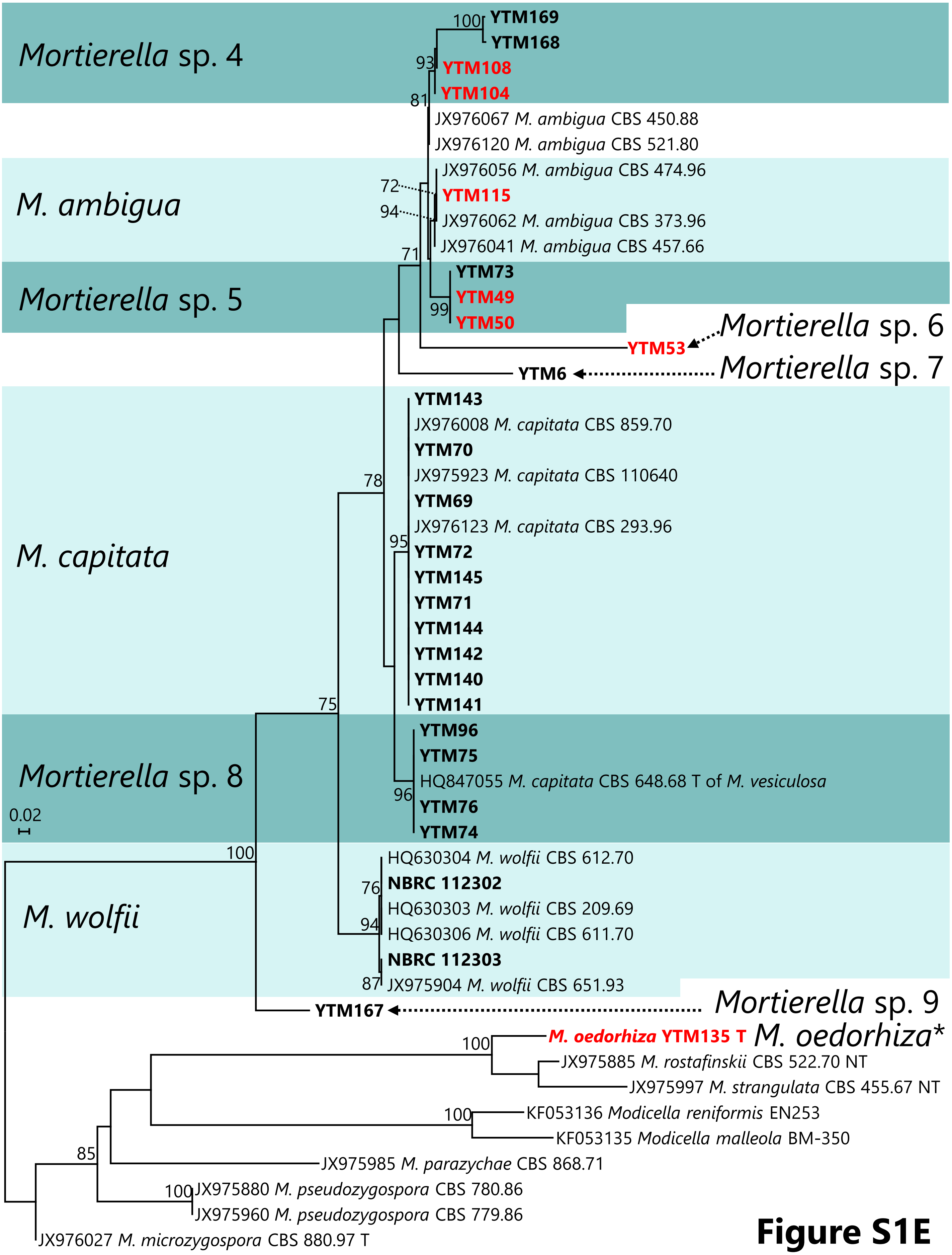
Mortierella sp. 4
100
YTM169
YTM168
93
YTM108
YTM104
81
JX976067 M. ambigua CBS 450.88
JX976120 M. ambigua CBS 521.80
M. ambigua
JX976056 M. ambigua CBS 474.96
72
YTM115
94
JX976062 M. ambigua CBS 373.96
JX976041 M. ambigua CBS 457.66
71
Mortierella sp. 5
YTM73
YTM49
99
YTM50
Mortierella sp. 6
YTM53
YTM6
Mortierella sp. 7
M. capitata
YTM143
JX976008 M. capitata CBS 859.70
YTM70
78
JX975923 M. capitata CBS 110640
YTM69
JX976123 M. capitata CBS 293.96
95
YTM72
YTM145
YTM71
YTM144
YTM142
YTM140
75
YTM141
Mortierella sp. 8
YTM96
YTM75
HQ847055 M. capitata CBS 648.68 T of M. vesiculosa
96
YTM76
0.02
YTM74
100
HQ630304 M. wolfii CBS 612.70
76
NBRC 112302
M. wolfii
HQ630303 M. wolfii CBS 209.69
94
HQ630306 M. wolfii CBS 611.70
NBRC 112303
87
JX975904 M. wolfii CBS 651.93
Mortierella sp. 9
YTM167
M. oedorhiza YTM135 T
M. oedorhiza*
100
JX975885 M. rostafinskii CBS 522.70 NT
JX975997 M. strangulata CBS 455.67 NT
100
KF053136 Modicella reniformis EN253
KF053135 Modicella malleola BM-350
85
JX975985 M. parazychae CBS 868.71
100
JX975880 M. pseudozygospora CBS 780.86
JX975960 M. pseudozygospora CBS 779.86
JX976027 M. microzygospora CBS 880.97 T

Figure S1E

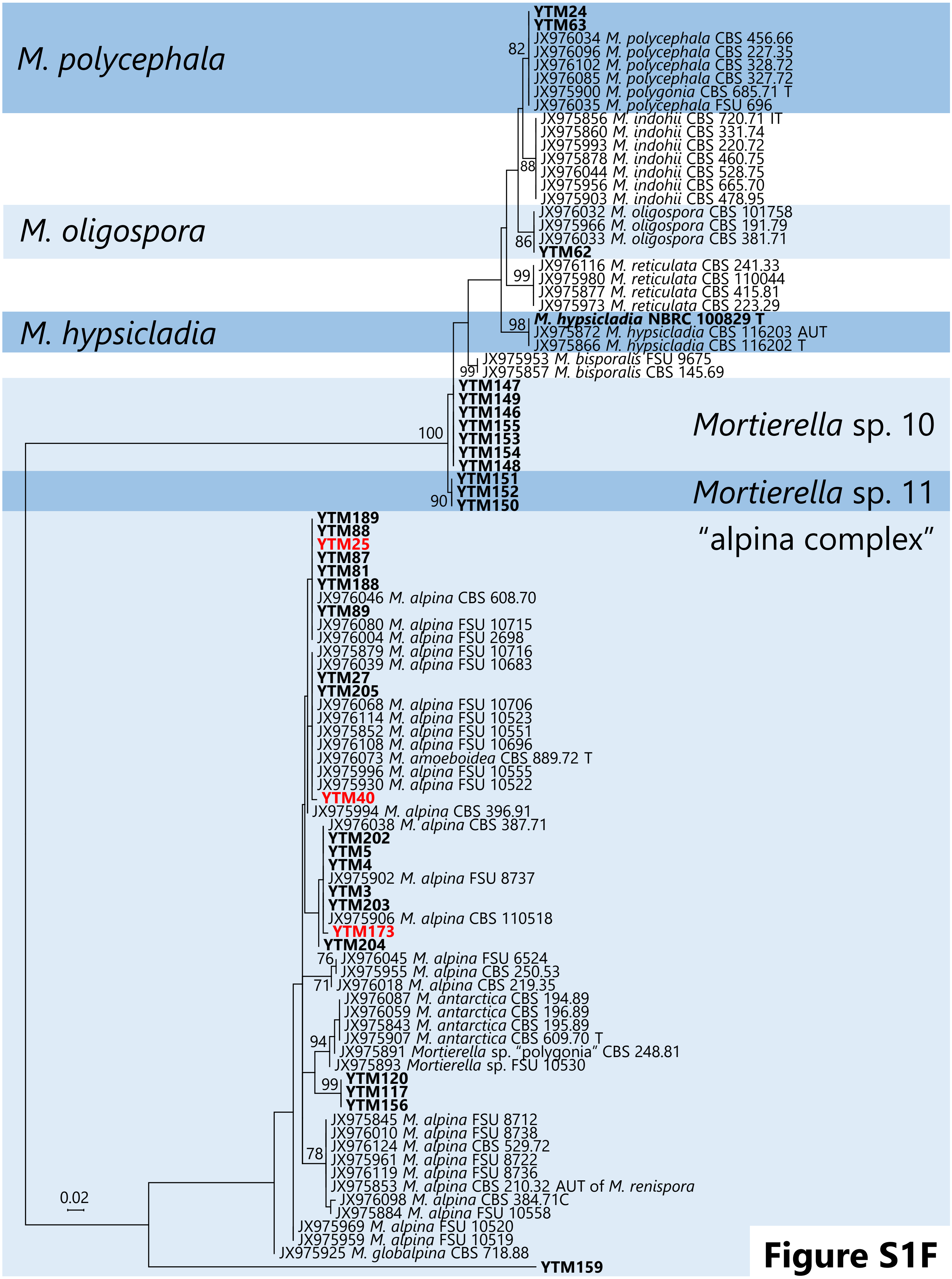
M. polycephala
YTM24
YTM63
JX976034 M. polycephala CBS 456.66
82
JX976096 M. polycephala CBS 227.35
JX976102 M. polycephala CBS 328.72
JX976085 M. polycephala CBS 327.72
JX975900 M. polygonia CBS 685.71 T
JX976035 M. polycephala FSU 696
JX975856 M. indohii CBS 720.71 IT
JX975860 M. indohii CBS 331.74
JX975993 M. indohii CBS 220.72
JX975878 M. indohii CBS 460.75
88
JX976044 M. indohii CBS 528.75
JX975956 M. indohii CBS 665.70
JX975903 M. indohii CBS 478.95
M. oligospora
JX976032 M. oligospora CBS 101758
JX975966 M. oligospora CBS 191.79
86
JX976033 M. oligospora CBS 381.71
YTM62
JX976116 M. reticulata CBS 241.33
99
JX975980 M. reticulata CBS 110044
JX975877 M. reticulata CBS 415.81
JX975973 M. reticulata CBS 223.29
M. hypsicladia
98
M. hypsicladia NBRC 100829 T
JX975872 M. hypsicladia CBS 116203 AUT
JX975866 M. hypsicladia CBS 116202 T
JX975953 M. bisporalis FSU 9675
99
JX975857 M. bisporalis CBS 145.69
YTM147
YTM149
YTM146
100
YTM155
YTM153
YTM154
YTM148
Mortierella sp. 10
YTM151
YTM152
90
YTM150
Mortierella sp. 11
"alpina complex"
YTM189
YTM88
YTM25
YTM87
YTM81
YTM188
JX976046 M. alpina CBS 608.70
YTM89
JX976080 M. alpina FSU 10715
JX976004 M. alpina FSU 2698
JX975879 M. alpina FSU 10716
JX976039 M. alpina FSU 10683
YTM27
YTM205
JX976068 M. alpina FSU 10706
JX976114 M. alpina FSU 10523
JX975852 M. alpina FSU 10551
JX976108 M. alpina FSU 10696
JX976073 M. amoeboidea CBS 889.72 T
JX975996 M. alpina FSU 10555
JX975930 M. alpina FSU 10522
YTM40
JX975994 M. alpina CBS 396.91
JX976038 M. alpina CBS 387.71
YTM202
YTM5
YTM4
JX975902 M. alpina FSU 8737
YTM3
YTM203
JX975906 M. alpina CBS 110518
YTM173
YTM204
76
JX976045 M. alpina FSU 6524
JX975955 M. alpina CBS 250.53
71
JX976018 M. alpina CBS 219.35
JX976087 M. antarctica CBS 194.89
JX976059 M. antarctica CBS 196.89
JX975843 M. antarctica CBS 195.89
94
JX975907 M. antarctica CBS 609.70 T
JX975891 Mortierella sp. "polygonia" CBS 248.81
JX975893 Mortierella sp. FSU 10530
99
YTM120
YTM117
YTM156
JX975845 M. alpina FSU 8712
JX976010 M. alpina FSU 8738
JX976124 M. alpina CBS 529.72
78
JX975961 M. alpina FSU 8722
JX976119 M. alpina FSU 8736
JX975853 M. alpina CBS 210.32 AUT of M. renispora
0.02
JX976098 M. alpina CBS 384.71C
JX975884 M. alpina FSU 10558
JX975969 M. alpina FSU 10520
JX975959 M. alpina FSU 10519
JX975925 M. globalpina CBS 718.88
YTM159

Figure S1F

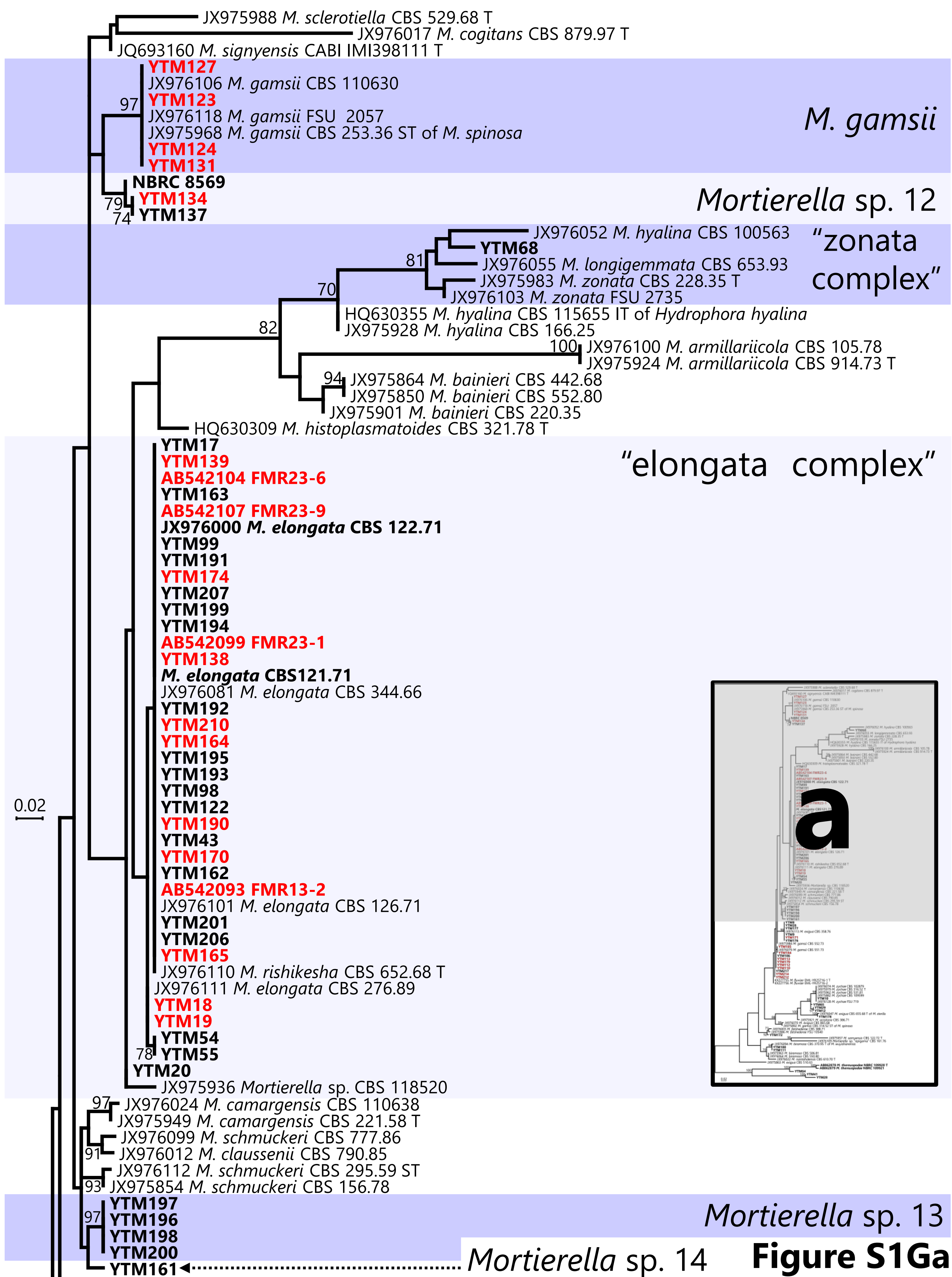
JX975988 M. sclerotiella CBS 529.68 T
JX976017 M. cogitans CBS 879.97 T
JQ693160 M. signyensis CABI IMI398111 T
YTM127
JX976106 M. gamsii CBS 110630
97
YTM123
JX976118 M. gamsii FSU 2057
JX975968 M. gamsii CBS 253.36 ST of M. spinosa
YTM124
YTM131
M. gamsii
NBRC 8569
79
YTM134
74
YTM137
Mortierella sp. 12
JX976052 M. hyalina CBS 100563
YTM68
81
JX976055 M. longigemmata CBS 653.93
JX975983 M. zonata CBS 228.35 T
JX976103 M. zonata FSU 2735
"zonata complex"
70
HQ630355 M. hyalina CBS 115655 IT of Hydrophora hyalina
82
JX975928 M. hyalina CBS 166.25
100
JX976100 M. armillariicola CBS 105.78
JX975924 M. armillariicola CBS 914.73 T
94
JX975864 M. bainieri CBS 442.68
JX975850 M. bainieri CBS 552.80
JX975901 M. bainieri CBS 220.35
HQ630309 M. histoplasmatoides CBS 321.78 T
"elongata complex"
YTM17
YTM139
AB542104 FMR23-6
YTM163
AB542107 FMR23-9
JX976000 M. elongata CBS 122.71
YTM99
YTM191
YTM174
YTM207
YTM199
YTM194
AB542099 FMR23-1
YTM138
M. elongata CBS121.71
JX976081 M. elongata CBS 344.66
YTM192
YTM210
YTM164
YTM195
YTM193
YTM98
YTM122
0.02
YTM190
YTM43
YTM170
YTM162
AB542093 FMR13-2
JX976101 M. elongata CBS 126.71
YTM201
YTM206
YTM165
JX976110 M. rishikesha CBS 652.68 T
JX976111 M. elongata CBS 276.89
YTM18
YTM19
YTM54
78
YTM55
YTM20
JX975936 Mortierella sp. CBS 118520
a
97
JX976024 M. camargensis CBS 110638
JX975949 M. camargensis CBS 221.58 T
JX976099 M. schmuckeri CBS 777.86
91
JX976012 M. claussenii CBS 790.85
JX976112 M. schmuckeri CBS 295.59 ST
93
JX975854 M. schmuckeri CBS 156.78
YTM197
97
YTM196
YTM198
YTM200
Mortierella sp. 13
YTM161
Mortierella sp. 14

Figure S1Ga

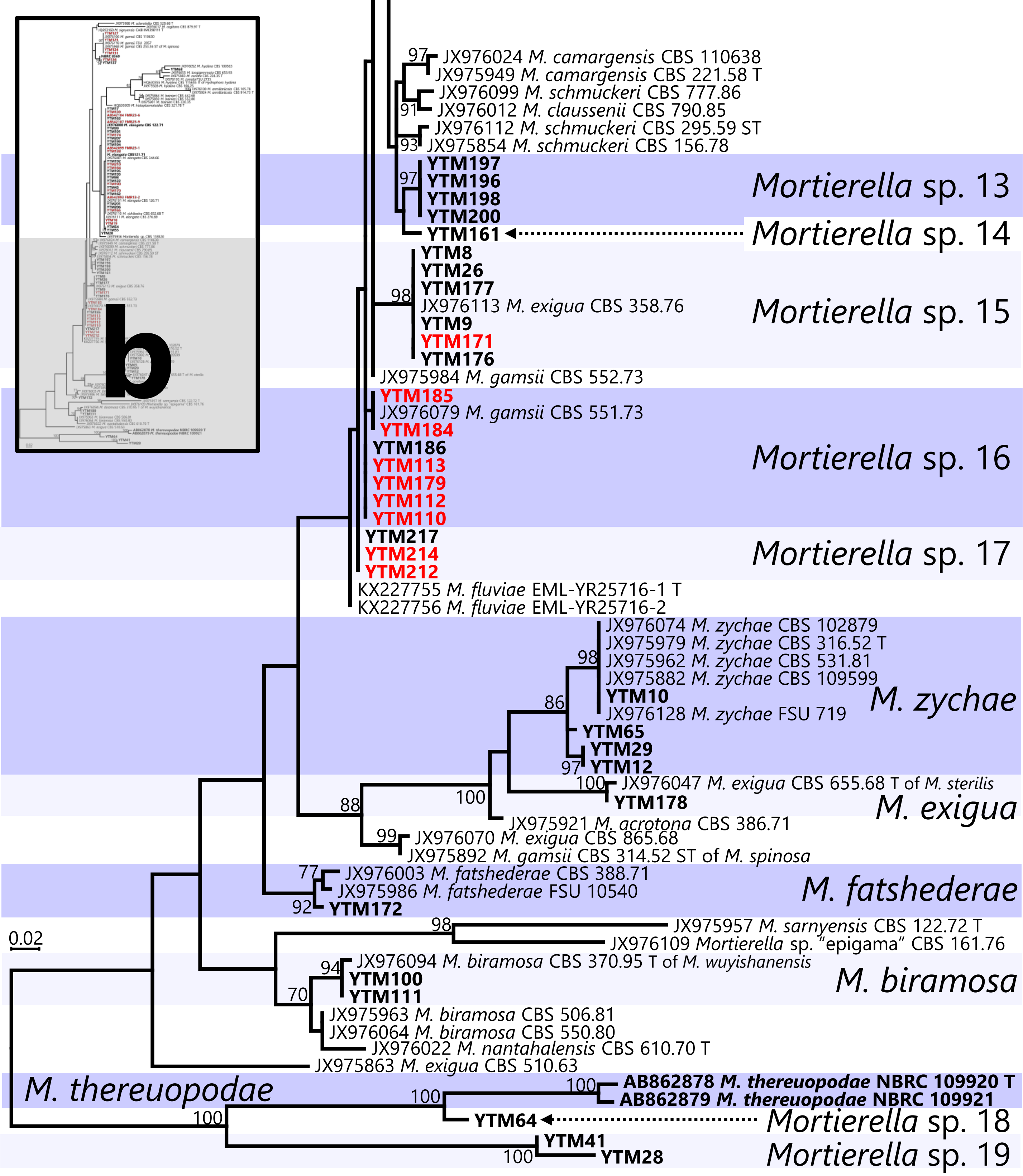
b
JX976024 M. camargensis CBS 110638
JX975949 M. camargensis CBS 221.58 T
JX976099 M. schmuckeri CBS 777.86
JX976012 M. claussenii CBS 790.85
JX976112 M. schmuckeri CBS 295.59 ST
JX975854 M. schmuckeri CBS 156.78
YTM197
YTM196
YTM198
YTM200
Mortierella sp. 13
YTM161
Mortierella sp. 14
YTM8
YTM26
YTM177
JX976113 M. exigua CBS 358.76
YTM9
YTM171
YTM176
Mortierella sp. 15
JX975984 M. gamsii CBS 552.73
YTM185
JX976079 M. gamsii CBS 551.73
YTM184
YTM186
YTM113
YTM179
YTM112
YTM110
Mortierella sp. 16
YTM217
YTM214
YTM212
Mortierella sp. 17
KX227755 M. fluviae EML-YR25716-1 T
KX227756 M. fluviae EML-YR25716-2
JX976074 M. zychae CBS 102879
JX975979 M. zychae CBS 316.52 T
JX975962 M. zychae CBS 531.81
JX975882 M. zychae CBS 109599
YTM10
JX976128 M. zychae FSU 719
M. zychae
YTM65
YTM29
YTM12
JX976047 M. exigua CBS 655.68 T of M. sterilis
YTM178
M. exigua
JX975921 M. acrotona CBS 386.71
JX976070 M. exigua CBS 865.68
JX975892 M. gamsii CBS 314.52 ST of M. spinosa
JX976003 M. fatshederae CBS 388.71
JX975986 M. fatshederae FSU 10540
YTM172
M. fatshederae
JX975957 M. sarnyensis CBS 122.72 T
JX976109 Mortierella sp. "epigama" CBS 161.76
0.02
JX976094 M. biramosa CBS 370.95 T of M. wuyishanensis
YTM100
YTM111
M. biramosa
JX975963 M. biramosa CBS 506.81
JX976064 M. biramosa CBS 550.80
JX976022 M. nantahalensis CBS 610.70 T
JX975863 M. exigua CBS 510.63
M. thereuopodae
AB862878 M. thereuopodae NBRC 109920 T
AB862879 M. thereuopodae NBRC 109921
YTM64
Mortierella sp. 18
YTM41
YTM28
Mortierella sp. 19

Figure S1Gb

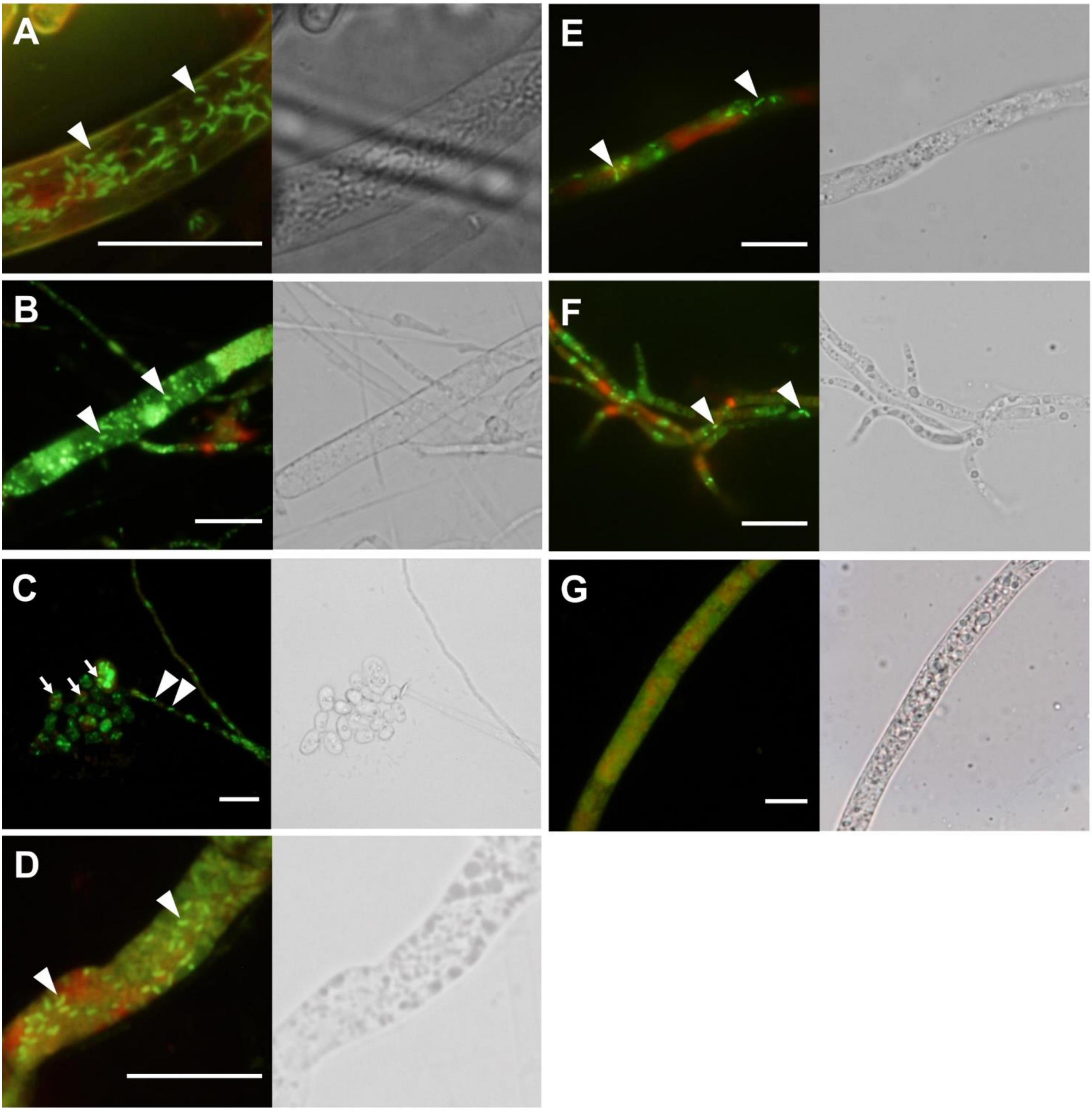

Figure S2
